# Disease related single point mutations alter the global dynamics of a tetratricopeptide (TPR) α-solenoid domain

**DOI:** 10.1016/j.jsb.2019.107405

**Published:** 2020-01-01

**Authors:** Salomé Llabrés, Maxim I. Tsenkov, Stuart A. MacGowan, Geoffrey J. Barton, Ulrich Zachariae

**Affiliations:** a Computational Biology, School of Life Sciences, University of Dundee, Dundee, UK; b Physics, School of Science and Engineering, University of Dundee, Dundee, UK

## Abstract

Tetratricopeptide repeat (TPR) proteins belong to the class of α-solenoid proteins, in which repetitive units of α-helical hairpin motifs stack to form superhelical, often highly flexible structures. TPR domains occur in a wide variety of proteins, and perform key functional roles including protein folding, protein trafficking, cell cycle control and post-translational modification. Here, we look at the TPR domain of the enzyme O-linked GlcNAc-transferase (OGT), which catalyses O–GlcNAcylation of a broad range of substrate proteins. A number of single-point mutations in the TPR domain of human OGT have been associated with the disease Intellectual Disability (ID). By extended steered and equilibrium atomistic simulations, we show that the OGT-TPR domain acts as an elastic nanospring, and that each of the ID-related local mutations substantially affect the global dynamics of the TPR domain. Since the nanospring character of the OGT-TPR domain is key to its function in binding and releasing OGT substrates, these changes of its biomechanics likely lead to defective substrate interaction. We find that neutral mutations in the human population, selected by analysis of the gnomAD database, do not incur these changes. Our findings may not only help to explain the ID phenotype of the mutants, but also aid the design of TPR proteins with tailored biomechanical properties.

## Graphical abstract

**Figure:**
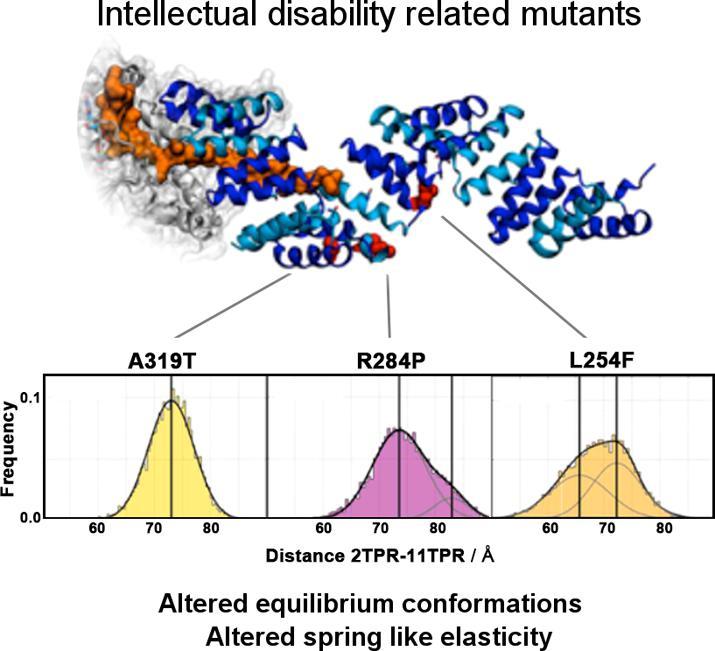

## 1 Introduction

Solenoid proteins represent ~5% of the human proteome. Due to their extended water-exposed surface and high degree of flexibility, they play a particularly important role in the formation of multiple protein–protein binding interactions. α-solenoid domains consist of arrays of repetitive α-helical units with variations in the numbers of repeats and the precise spatial arrangement of the helices (Kobe and Kajava, 2000). Globally, most α-solenoid domains adopt extended superhelical shapes. The most common types of repetitive α-helical units are tetratricopeptide (TPR), HEAT, armadillo and leucine-rich repeats. Each repeat type possesses a characteristic conserved sequence of amino acids, which determines the specific fold of the units and influences the geometry and dynamics of the entire domain (Kajava, 2012).

In the case of HEAT repeat domains, simulations have previously shown that the conserved hydrophobic core formed by part of this consensus sequence confers fully reversible, spring-like elasticity to the domains (Kappel et al., 2010). Armadillo repeat domains, by comparison, are more rigid, although they are still sufficiently flexible to accommodate a range of different binding partners (Pumroy et al., 2015). The computationally predicted nanospring behaviour of alpha-solenoid domains has been experimentally confirmed for a designed protein consisting of three TPR repeats (Cohen et al., 2015). Altogether, it appears likely that the consensus sequence of the repetitive units governs the global dynamics of the domain. Depending on the specific functional role of the domain, this enables fine-tuning of its flexibility, while retaining stability against unfolding (Cohen et al., 2015, Mejías et al., 2016).

The TPR consensus sequence contains 34 residues, in which the conserved positions are W_4_-L_7_-G_8_-Y_11_-A_20_-F_24_-A_27_-P_32_ (Goebl and Yanagida, 1991, Sikorski et al., 1990). The sequence folds into a characteristic α-helix-turn-α-helix (helices A and B) motif (D’Andrea and Regan, 2003). TPR domain proteins are involved in a wide range of cellular processes such as protein folding (Blatch and Lässle, 1999, D’Andrea and Regan, 2003, Das et al., 1998, Taylor et al., 2001), cell cycle control (Sikorski et al., 1991), post-transcriptional modification (Gundogdu et al., 2018) and mitochondrial and peroxisomal protein transport (Chan et al., 2006, Fodor et al., 2015). Evolutionarily, the domains are likely to have arisen from the amplification of an ancestral helical hairpin structure (Zhu et al., 2016). Next to naturally occurring TPR domains, engineered TPR proteins have recently gained substantial interest as they allow the design of optimised protein–protein assembly surfaces (Cortajarena et al., 2008, Sanchez-deAlcazar et al., 2018).

**Fig. 1:**
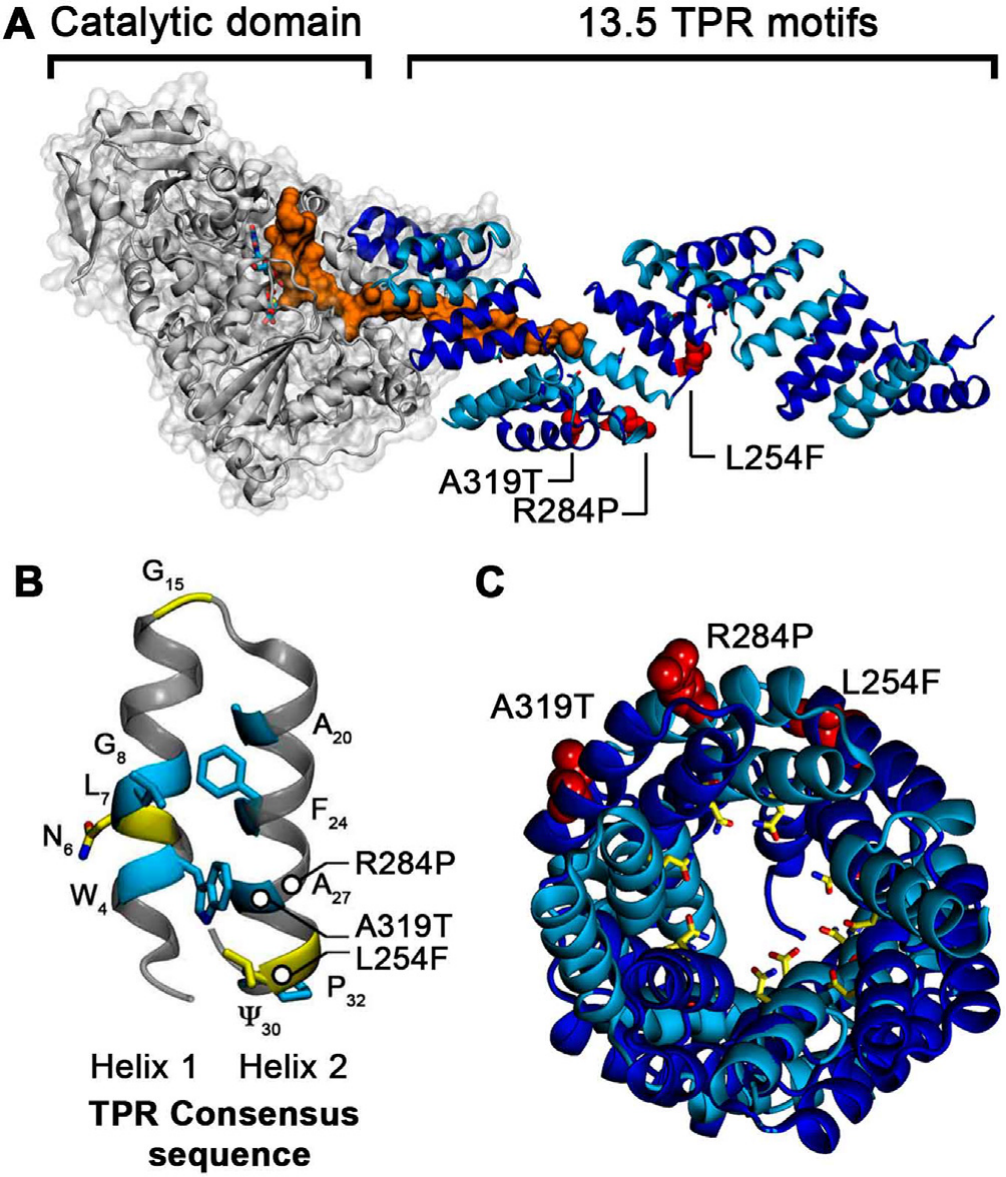
Superhelical structure of the OGT-TPR domain and location of the ID-associated mutation sites. (A) Structure of the OGT enzyme with the catalytic domain in grey and the TPR domain in blue cartoon representation. A short peptide belonging to a substrate protein, TAB1, is shown bound to OGT in orange (PDB id: 5LVV) (Rafie et al., 2017). The ID-associated mutations L254F, R284P and A319T are shown as red spheres. (B) TPR consensus sequence (CS) with the most conserved residues shown as blue sticks. The OGT enzyme contains additional conserved residues in its CS, shown as yellow sticks. (C) View of the central tunnel shaped by the TPR superhelix, thought to form the major interaction surface for substrate proteins. The conserved N_6_ asparagine residues of the OGT-TPR domain, which are the main substrate interaction sites, are shown in yellow; the locations of the single point mutations are shown in red.

O-linked GlcNAc-transferase (OGT; Fig. 1A) is a TPR-domain containing enzyme that catalyses O–GlcNAcylation, a reversible post-transcriptional modification of protein substrates including transcription factors and cytoskeletal proteins (Iyer and Hart, 2003). The OGT-TPR domain recognises and binds substrate proteins and must therefore be able to adapt to a wide range of different protein sizes and geometries (Jínek et al., 2004). This capacity is shared with other α-solenoid domains, such as the HEAT and armadillo repeat domains that bind cargo proteins as nuclear transport receptors (Chook and Süel, 2011, Stewart, 2007). The OGT-TPR domain possesses an extended consensus sequence (N_6_-L_7_-G_8_-G_15_-A_20_-Y_24_-A_27_-Ψ_30_-P_32_), which includes three additional positions compared to most other TPR repeats (Zeytuni and Zarivach, 2012) (Fig. 1B). Three single point mutations that are associated with Intellectual Disability (ID) phenotypes are located within repeat units TPR7 (L254F), TPR8 (R284P) and TPR9 (A319T), far from the catalytic domain of the enzyme (Willems et al., 2017) (Fig. 1A,C). Intellectual disability is a disease which leads to an early-onset impairment of cognitive function and the limitation of adaptive behaviour (Ropers, 2010). The X-ray structures of both the wild-type protein (wt, PDB id: 1W3B) (Jínek et al., 2004) and the ID-associated OGT mutant L254F (PDB ID 6EOU) (Gundogdu et al., 2018), have recently been determined.

Here, we were interested to investigate both the global domain flexibility of the wt OGT-TPR domain as well as the effects of the ID-related single point mutations. We therefore conducted microsecond all-atom molecular dynamics simulations, both unbiased and steered, of wt and mutant OGT proteins and analysed the effect of the mutations on the dynamic properties of the domain. Our simulations first establish the TPR domain as an elastic nanospring. Furthermore, our results show that each of the single mutations alters the conformational dynamics of the domain in a different way, and leads to distinct changes in the overall biomechanical properties of OGT-TPR, while all of them display a strong divergence from the wt. These modified dynamics may play an important role in the capacity of the OGT enzyme to bind its various substrate proteins and therefore help to explain the ID phenotype of these mutants. Moreover, our findings may provide information how engineered TPR proteins could be conferred with fine-tuned dynamic properties during their structural design.

## 2 Results & discussion

### 2.1 The OGT-TPR domain is a protein nanospring with fully reversible elasticity

To characterise the elasticity of the OGT-TPR domain and to ascertain if its folded structure remains intact upon enforced elongation, we performed steered molecular dynamics (SMD) simulations on wild-type (wt) OGT-TPR (Jínek et al., 2004) and the ID-associated mutants (Gundogdu et al., 2018). The available OGT-TPR crystal structures include sequence positions 26–410, comprising ten complete TPR units (TPR_2–11_) and two partially resolved repeats. We used a moving harmonic potential of ~1.25 kcal mol^−1^ Å^−2^, attached to the C-terminal end of the TPR domain (TPR11), at a velocity of 1 Å ns^−1^ to increase its separation from the fixed N-terminus (TPR2) and thereby elongate the domain. We then extracted four independent extended conformations obtained from the trajectory under force and allowed the OGT-TPR domain to relax its conformation in further unbiased simulations.

**Fig. 2:**
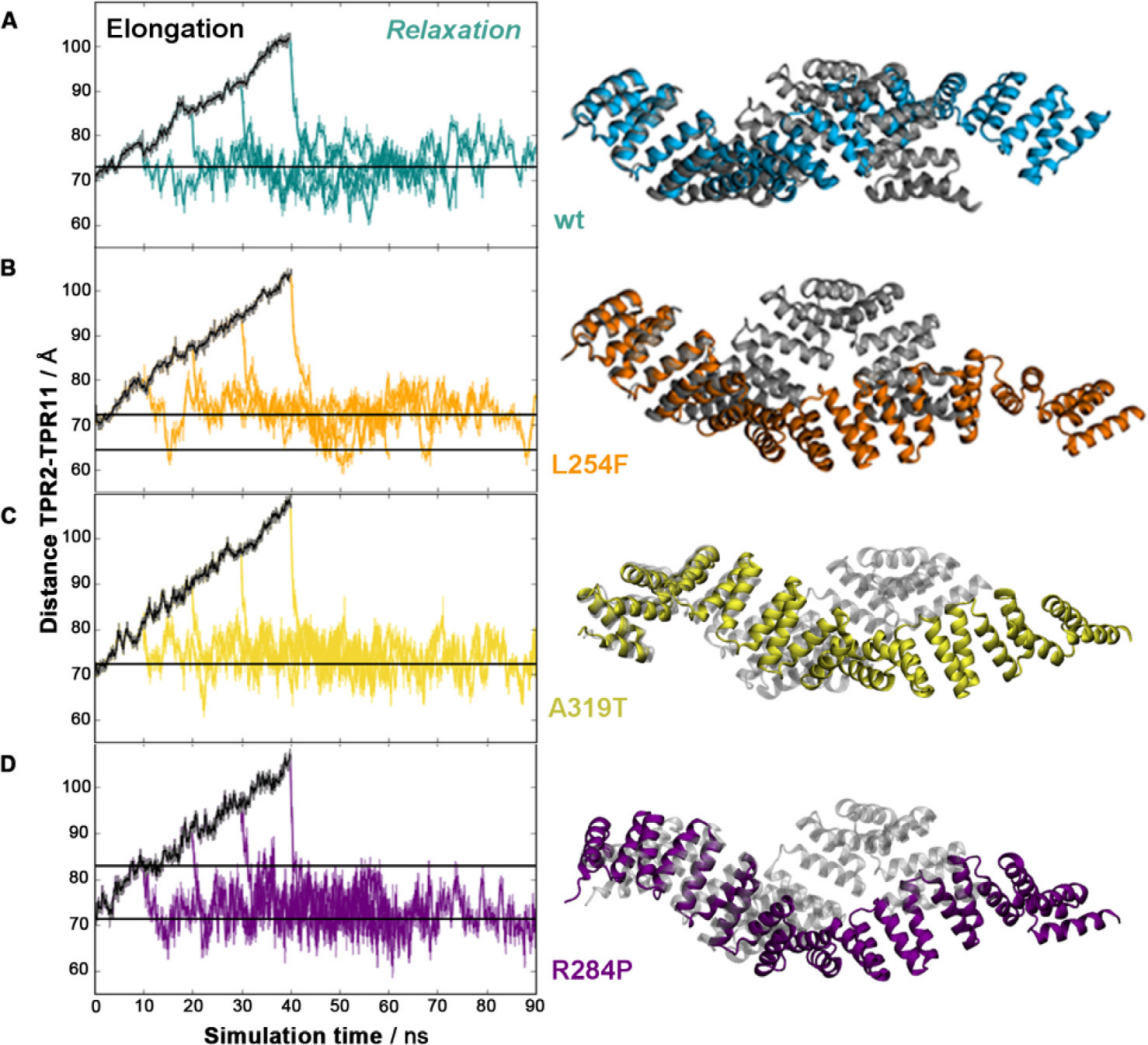
Elasticity of the OGT-TPR domain. (A, left) Elongation of the wt domain under steered MD simulations (black) and subsequent relaxation of the domain back to its original length starting from four different points on the elongation trajectory (teal). (A, right) Maximally elongated state from which elastic relaxation to the original conformation is observed (teal) and comparison to the starting structure (grey). (B, C, D) Reversible elasticity and maximum elongation of the mutants L254F (orange), A319T (yellow), and R284P (purple).

As shown in Fig. 2A, all of the elongated conformations of wt OGT-TPR relax back to their original end-to-end distance on very short timescales, only spanning few ns. Although the fluctuation level around the equilibrium distance is relatively high (reflecting the high flexibility of the TPR domain), the final states regain conformations close or even identical to the original domain extension observed in the crystal structure. We thus find that the wt TPR domain shows fully reversible elasticity up to elongations of ~145% of its original length. During expansion, no rupture events occur that might affect the intramolecular contacts that are essential for maintaining its structure. This level of elasticity is similar to the elastic behaviour previously described for the HEAT repeat protein importin-β (Kappel et al., 2012, Kappel et al., 2010).

The results thus show that the OGT-TPR superhelix displays spring-like mechanical behaviour. Enforced extensions or distortions of the structure lead to a loading of this protein nanospring, by which energy is stored in the elongated conformation without disrupting its secondary structure or intramolecular contacts. Upon release of the driving force, the elongated superhelix elastically relaxes to its original ground state, thereby releasing the energy that was previously stored.

SMD simulations of the ID-related mutants (Gundogdu et al., 2018, Willems et al., 2017) (Fig. 2B-D) show that these mutations do not disrupt the elastic spring behaviour of the domain. In fact, the ID variants can sustain slightly larger end-to-end extensions in the fully elastic regime before disruption of the secondary structure occurs. The maximum extensions we observe for the mutants are ~103.7 Å (L254F), 107.5 Å (A319T), and ~ 107.2 Å (R284P), compared to ~101.7 Å for the wt. Like the wt, all of the variants relax back to their original end-to-end length after release of the driving force. This suggests that the principal spring-like behaviour of the domain is robust against these single-point changes. We therefore conclude that the mutations do not incur a globally misfolded domain structure but rather lead to more subtle changes in the dynamical and biomechanical properties of the domain, which will be most relevant for protein–protein binding interactions. Support for this notion comes from recent experiments, in which only moderate deviations from the melting temperature of the wt domain were observed for these mutants (Selvan et al., 2018).

## 3 Biomechanical properties of the OGT-TPR domain and effect of ID-related mutations

**Fig. 3:**
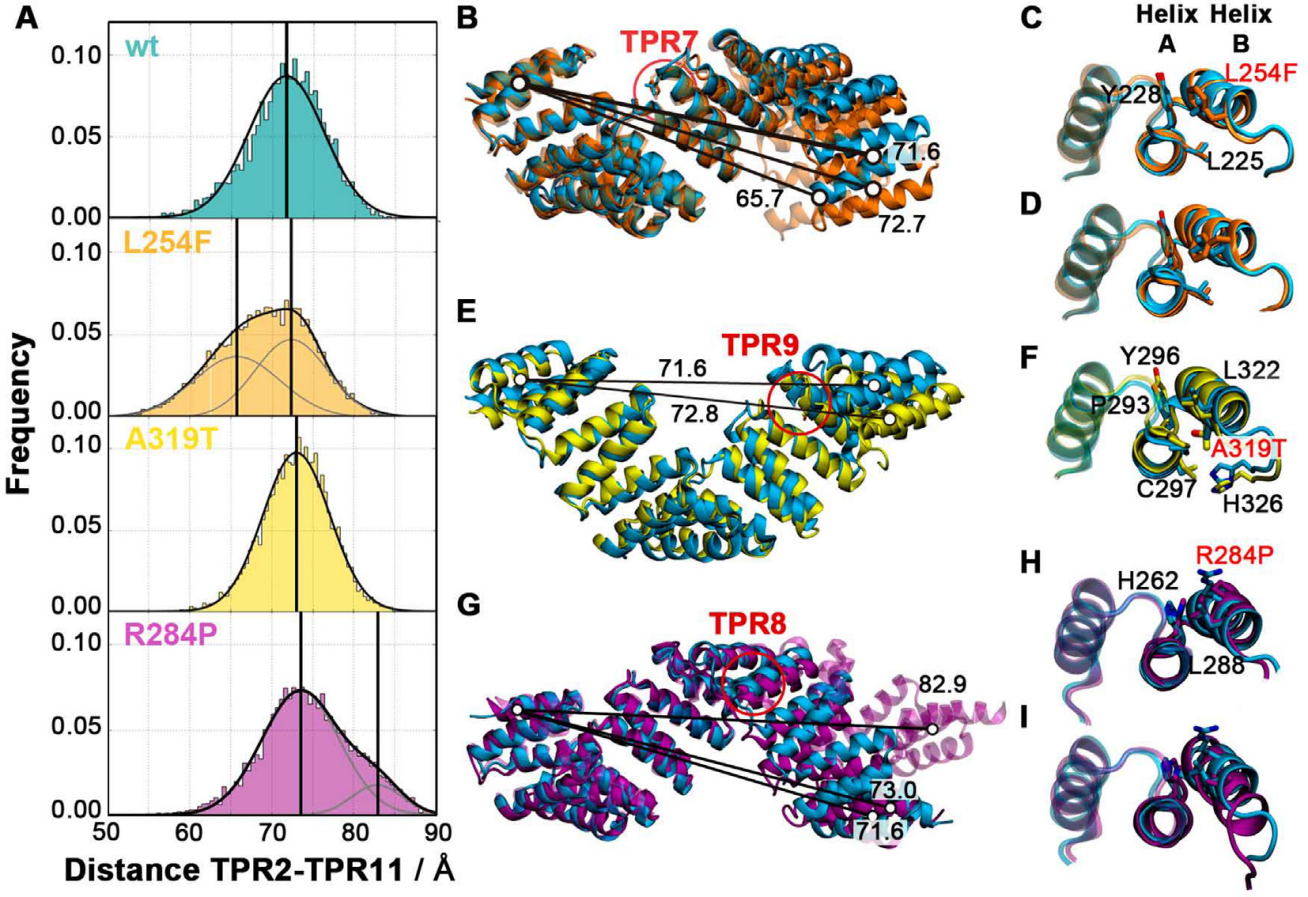
Effect of the ID-related single mutations on the elasticity of the TPR domain and local conformational changes. (A) End-to-end distance of the OGT-TPR domain of the wt (cyan), L254F mutant (orange), A319T mutant (yellow) and R284P mutant (purple). (B, C, D) Superposition of the representative equilibrium conformations of the wt (cyan) with the L254F (orange), A319T (yellow) and R284P mutant TPR-OGT domains, respectively. Indicative distances (Å) between the centres-of-mass of TPR2 and TPR11 are shown for the major mutant conformations; the mutation site is highlighted. (E) Representative snapshots of the two major conformations of TPR7 and the F254 sidechain compared to L254 of the wt domain. (F) Representative snapshot of TPR9 and the T319 sidechain compared to A319 of the wt domain. (G) Representative snapshots of the two major conformations of TPR8 and the P284 sidechain compared to R284 of the wt domain.

To accurately determine the spring constant of the domain and thereby obtain the energy required for its elastic deformation, we conducted further equilibrium MD simulations. The wt and mutant OGT-TPR domains were each simulated for a total time of 2 µs, combining data from four replicates of 500 ns length. Fig. 3A shows the distribution of end-to-end (TPR2-11) domain distances observed during the simulations.

In the case of the wt, the extensions are normally distributed, reflecting the fluctuations of the TPR domain around a single equilibrium length of ~71.6 Å. Since the fluctuations of a spring are related to the spring constant by *k_spring_ = k_B_T / σ^2^*, the width *σ* of the normal distribution (its standard deviation) gives rise to a spring constant of *k_WT_* = 20.80 ± 0.07 pN/nm (Table 1). For comparison, the spring constant found for the HEAT repeat domain of importin-β is ~10 pN/nm (Kappel et al., 2010), while that of the armadillo-repeat domain of importin-α lies between 80 and 120 pN/nm (Pumroy et al., 2015). The spring constant of OGT-TPR signifies that an extension or compression of the OGT-TPR domain by 1 nm requires an energy input of ~6 kJ/mol, while an energy of ~ 55 kJ/mol is necessary to obtain the maximum elastic extension we observe in our steered simulations. Upon binding and accommodating substrate proteins of different size, the energy for the distortion of the superhelix is likely provided by the binding energy of the substrate to the TPR domain.

**Table 1:** Spring constants of the studied OGT-TPR variants.

OGT-TPR variant	Spring constant (pN/nm)	Phenotypic effect
Wild-type	20.80 ± 0.07	–
L254F major conformation	21.99 ± 0.14	ID-related
L254F minor conformation	12.61 ± 0.08	ID-related
R284P major conformation	19.92 ± 0.01	ID-related
R284P minor conformation	17.02 ± 0.13	ID-related
A319T	24.90 ± 0.11	ID-related
A310T	20.31 ± 0.09	neutral
I279V	21.40 ± 0.07	neutral
L254I	22.99 ± 0.08	unknown

Importantly, this elasticity provides the domain with the ability to transiently store part of this binding energy in the form of distorting the superhelix and release this energy upon substrate dissociation. In HEAT repeat proteins, the capacity to store binding energy has been identified as a crucial factor that aids in accelerating the disassembly of protein–protein complexes. The extended surface of α-solenoid domains optimises binding selectivity by providing a multitude of specific binding interactions, whose sum however also leads to very large protein–protein binding energies (Lee et al., 2005, Zachariae and Grubmüller, 2008). Protein complexes of such high affinity would show exceptionally slow off-rates upon disassembly, unless some of the substrate binding energy could be stored in the deformation of the α-solenoid superhelix. In this way, extended α-solenoid domains are likely to fine-tune their protein–protein binding thermodynamics (Lee et al., 2005, Zachariae and Grubmüller, 2008, Zachariae and Grubmüller, 2006).

The distributions of end-to-end distances of the ID-related domain variants are displayed in Fig. 3A. In contrast to the wt, the L254F variant shows a partition into two populations with different average extension. The main population has an average extension of ~72.7 Å, while a second Gaussian distribution is observed around ~65.7 Å. The end-to-end distances of the R284P mutant domain also display a separation into two populations. Here, the main population has an extension similar to the wt (~73.0 Å), while the secondary population shows an increased length of ~82.9 Å. The end-to-end distance distribution of the A319T variant does not display a separation into sub-populations, and its mean remains near the wt value (~72.8 Å). However, the width of the normal distribution is markedly reduced compared to wt, indicating a modification of its nanospring behaviour. We therefore derived the spring constants for all the major conformational populations of the mutants.

The major species of the L254F mutant has a spring constant similar to the wt (*k_L254F1_* = 21.99 ± 0.14 pN/nm) while the shorter population shows a markedly softened spring constant of *k_L254F2_* = 12.61 ± 0.08 pN/nm. For the R284P mutant, the lengthened population also reflects a softer nanospring with a spring constant of *k_R284P2_* = 17.02 ± 0.13 pN/nm, while the major population remains close to the wild-type (*k_R284P1_* = 19.92 ± 0.01 pN/nm). By contrast, the narrower distribution of lengths observed for the A319T variant emerges due to a rigidified nanospring (*k_A319T_* = 24.90 ± 0.11 pN/nm).

These results show that all of the ID-related single point mutations in the TPR domain induce substantial changes in the biomechanical properties of the domain. The malleability of the domain, and its capacity to adapt to different substrate proteins, is key to enabling the function of the OGT enzyme, however. Furthermore, as shown previously for HEAT repeat proteins (Lee et al., 2005, Zachariae and Grubmüller, 2008), the nanospring character of α-solenoid domains is crucial for the reversibility of protein–protein binding during substrate release by enabling the transient storage and release of binding energy. Our finding that all of the ID-related OGT-TPR mutants exhibit a significant alteration in their spring-like behaviour thus indicates likely defects in their capacity to bind and efficiently release substrates.

## 4 Local conformational effects propagate into globally altered L254F and R284P states

While all the mutations lead to a substantial modification of the spring constant of the OGT-TPR domain, two mutants, L254F and R284P, additionally show populations that deviate from the overall wt equilibrium length. We were therefore interested how these single-point mutations propagate into a global conformational change of the domain. We monitored the local geometry around the mutated site using three structural determinants of the individual repeats (see Fig. S2 for a graphical representation): the intra-TPR distance (distance between the Cα atoms of TPR unit positions Ψ1 and Ψ30), the inter-TPR distance (distance between the centres of mass of consecutive repeats), and the angle formed by the Cα atoms of position Ψ_30_ of the previous repeat and the positions Ψ_1_ and Ψ_30_ of the mutated TPR repeat (B-A’-B’ angle, Fig. S2) (Gundogdu et al., 2018). This angle quantifies the turn between repeats, which contributes to the formation of the global TPR superhelix. As measures of the global domain conformation, we used the end-to-end distance of the domain, as before, as well as its root mean square deviation (RMSD) during the simulations.

**Fig. 4:**
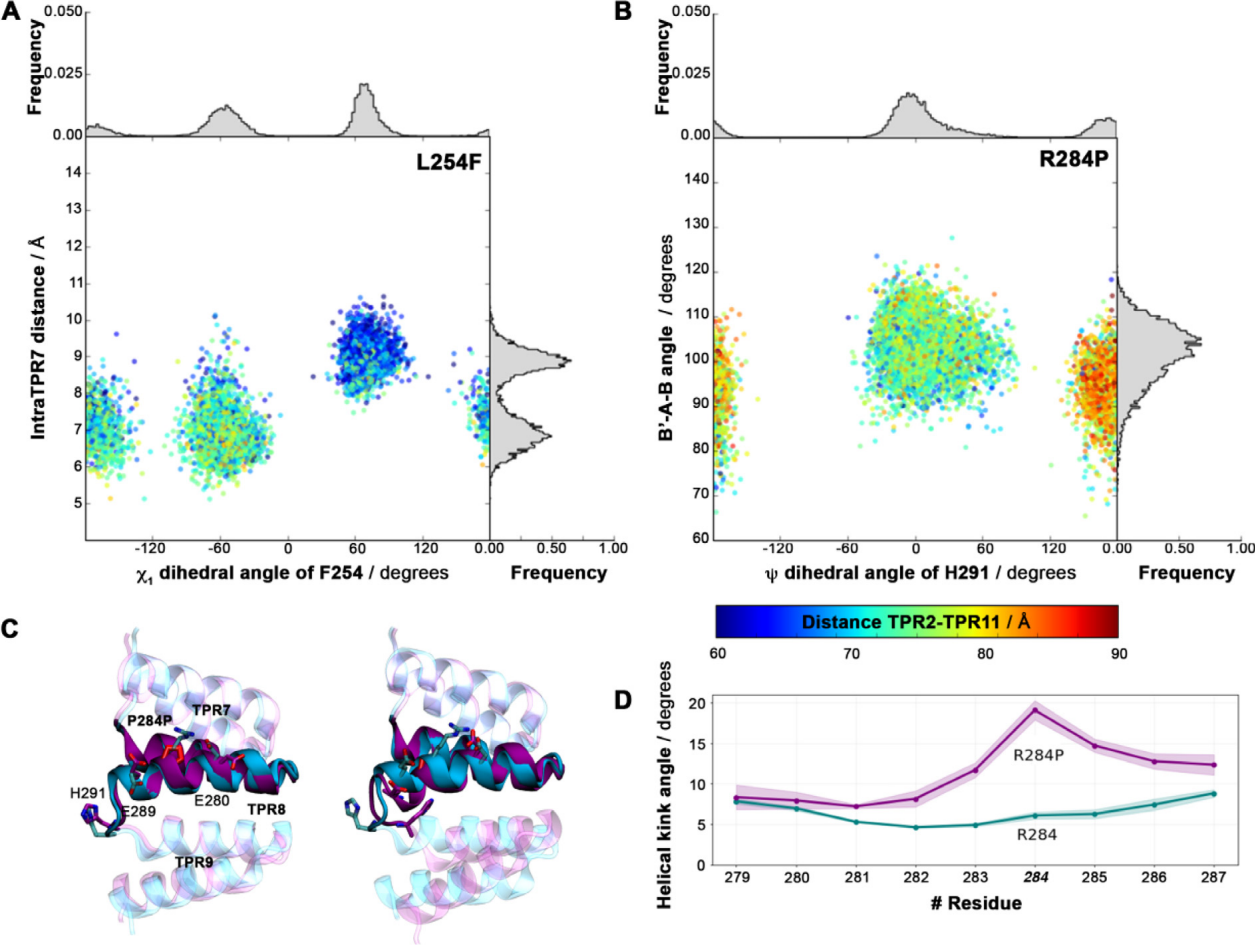
Local conformational populations of the L254F and R284 mutants. (A, B) Major local structural determinants are shown on the x- and y-axis; the global TPR2–TPR11 distance is colour-coded. A clear correlation between the local conformation and the global length is seen. (A) The x-axis displays the X1 dihedral angle of residue F254, the y-axis shows the intra-TPR7 distance; normalised histograms in grey show the relative distributions of the local populations. (B) The ψ dihedral angle of residue H291 is shown on the x-axis, the angle between consensus position 30 of TPR8 and positions 2 and 30 of TPR9 (B’-A–B) on the y-axis; normalised histograms display the relative distributions of the populations. (C) Representative conformations of the two populations found for R284P. The R284P and wt domains are shown in purple and cyan cartoon and sticks, respectively. (C) Kink angle between successive residues in TPR8 helix B for R284P (purple) and wt (cyan).

In the wt domain, TPR7 shows an intra-TPR7 distance of ~6.63 ± 0.39 Å and a 6B-7A-7B angle of 107.80° ± 4.20°. The L254 side chain is buried between TPR7 helices A and B, establishing van der Waals interactions with the side chains of L225 and Y228. Its first side chain dihedral angle, χ1, adopts a single conformation at −72.24° ± 12.73°. By contrast, the bulkier Phe side chain in the L254F mutant can adopt three conformations around this dihedral angle – two major orientations (with χ_1_ = -54.51° ± 13.94°, termed LF1, and χ_1_ = 69.58° ± 9.74°, termed LF2, shown in Fig. 3E) as well as a transient state (χ_1_ = -167.53° ± 12.19°, LF3), as previously reported in Gundogdu et al. (Gundogdu et al., 2018) (Fig. 4A). In both the wt and L254F crystal structures, only the LF1 conformation is seen. In the mutant LF1 conformation, the phenyl moiety interacts with the side chains of L225, Y228 and R245. The wt B6-A7-B7 angle is maintained (113.53° ± 5.51°), while the intra-TPR7 distance (6.83 ± 0.50 Å) remains close to the wt (Fig. 4A, S9). The LF2 conformation of the mutant shows an increase in the intra-TPR7 distance (to 8.83 ± 0.38 Å) and a reduced B6-A7-B7 angle (93.88° ± 4.91°). These local conformational changes enable the phenyl moiety of the mutant to wedge in between the TPR7 helices and interact with the side chains of N224, L225 and Y228 (Fig. 3E). The two major different local conformational states within mutant repeat TPR7 propagate to the neighbouring repeat modules and, as a consequence, modify the overall geometry of the domain. The global end-to-end distance distribution of the L254F mutant is thus bimodal, with two Gaussians reflecting the two major conformations of the F254 residue (Figs. 4 and 3A). In the case of the A319T mutation, we find that the rigidification of the nanospring is due to the formation of an additional hydrogen bond between the side chain of T319 on TPR9 helix B and the backbone of Y296 on TPR9 helix A (Fig. 3F).

The R284P mutation, located in repeat 8 (TPR8) at position X_26_ outside the TPR consensus sequence, introduces a proline residue in the middle of TPR helix B. This mutation restricts the mobility of the sidechain and abolishes the salt bridge between R284 and residues E280 and E289 from the same helix. Additionally, a proline residue cannot establish the wt hydrogen bond with the previous helix turn, distorting the helical domain. This increases the distance between the backbone O atom of E280 and the N atom of the P284 side chain from 3.00 ± 0.16 Å in the wt to 4.62 ± 0.25 Å in the mutant. Additionally, in a minor population, the helix develops a kink of 19.1 ± 1.0° (Figs. 3G, 4D). The altered geometry of TPR8 influences the neighbouring TPR unit by changing the inter-repeat angle and modifying the conformation of residue H291 on TPR9 (Fig. 4B**)**. In the R284P minor conformation, the H291 side chain resides between the side chain of F292 and the backbone of the A285, while it is solvent-exposed in both the wt and the major conformation of R284P (Fig. 4C). The substantial local rearrangements in the smaller population of R284P are then propagated into a globally elongated domain conformation.

## 5 Biomechanical properties of neutral OGT-TPR mutations

To differentiate mutations that lead to pathological phenotypes from neutral mutations that are observed in humans but are not related to disease, we selected two further OGT-TPR variants (I279V and A310T), which are unlikely to lead to aberrant OGT function. In addition, we probed an alternative OGT-TPR variant at position 254 (L254I) to investigate if other mutations at this site give rise to a distortion of the TPR nanospring similar to that observed for L254F (Fig. 3). L254F results in a partition of the global conformational ensemble into two populations with differing overall extension. The three mutants were each subjected to simulations of 2 µs total length.

Candidates for control mutagenesis unlikely to cause disease phenotypes were selected from OGT variants present in the Genome Aggregation Database (gnomAD) (Karczewski et al., 2019). The gnomAD variants are less likely to be disease-associated since they are derived from healthy individuals with no serious disease phenotypes. We considered that variants observed in at least one male would make good control candidates because OGT is located on the X chromosome and OGT disorders are X-linked (Table S1). This distinguished nine of the 22 gnomAD variants as ideal control candidates. Amongst these, variants I279V and A310T are located in repeats with known pathogenic variants (i.e., TPRs 8 and 9, respectively) and on this basis were considered to be relevant choices for this study. Although these nine variants present in males are similarly conservative in terms of residue physicochemical properties, the selected I279V and A310T are amongst the most conservative substitutions on the Zvelebil scale (Zvelebil et al., 1987). Also, I279V is the most common OGT-TPR missense variant in gnomAD overall, providing further evidence that it is unlikely to have significant deleterious effects. Finally, residues I279 and A310 are unconserved with respect to an alignment of human Swiss-Prot TPR domains of canonical length (34 amino acids), annotated by SMART and obtained via InterPro (Letunic and Bork, 2018, Mitchell et al., 2019).

**Fig. 5:**
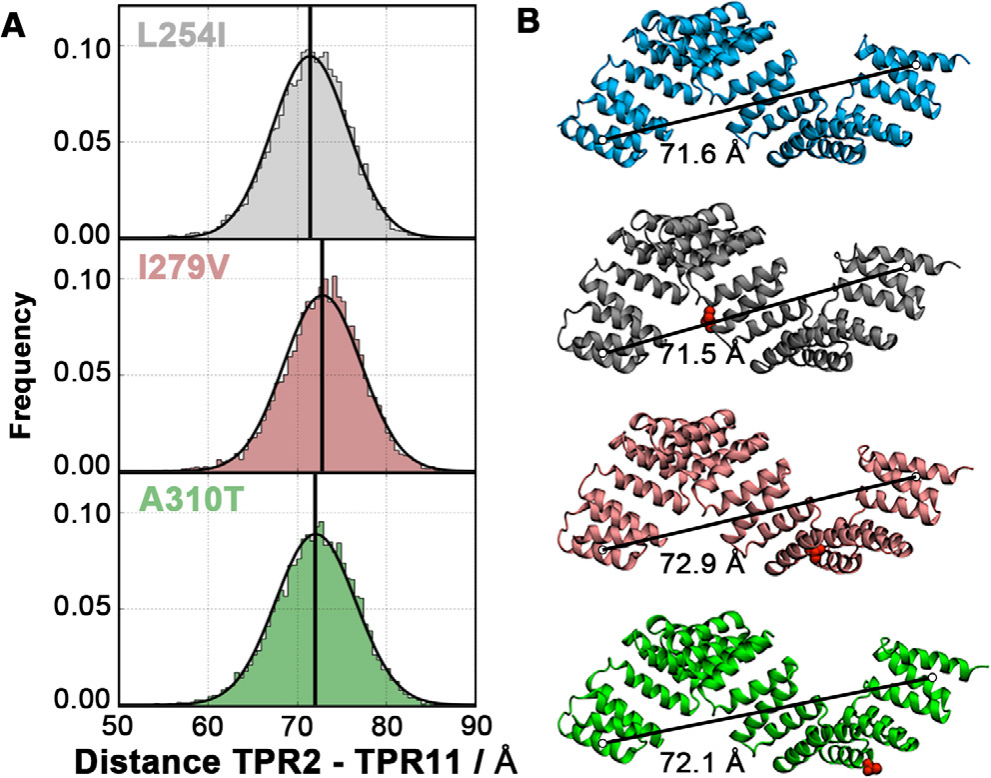
Effect of the neutral single point mutations on the elasticity of the TPR domain. A) End-to-end distance of the OGT-TPR domain of the L254I mutant (grey), A310T mutant (green) and I279V mutant (pink). B) Equilibrium structures of the wild-type and negative control OGT-TPR domains. The wild-type, L254I, I279V and A310T TPR domains are shown as blue, grey, pink and green cartoon respectively. Mutations are highlighted in red spheres. The figure also shows the equilibrium end-to-end distances between TPR2 and the TPR11.

As seen in Fig. 5, the two variants selected by this procedure (I279V and A310T) show no distortion of the TPR nanospring. The dynamic populations of each of these neutral mutations display single distributions centred around the length of the wt TPR domain, with average extensions of ~72.9 Å and ~72.1 Å for the I279V and A310T mutant domains. The domain conformations are therefore identical to that adopted by the wt TPR domain (Fig. 5B and S15). Furthermore, the spring constants derived from equilibrium fluctuations of the TPR nanospring remain close to the wt with *k_I279V _*= 21.40 ± 0.07 pN/nm (I279V) and *k_A310T_* = 20.31 ± 0.09 pN/nm (A310T). These results show that the neutral mutations neither alter the global conformation nor the biomechanical behaviour of the OGT-TPR domain.

For L254I OGT-TRP, our simulations also show that this variant does not exhibit any distortions of the TPR domain, in contrast to the disease-associated mutant L254F. The end-to-end length of the L254I TPR domain displays a single distribution around the wt extension of ~71.5 Å. The spring constant of this mutant shows a slight increase compared to the wt value with *k_L254I_* = 22.99 ± 0.08 pN/nm (Table 1), albeit less than the ID-related mutant A319T. It is important to note here, however, that the variant L254I has neither been described to cause ID phenotypes nor can it be ruled out to show any deleterious behaviour according to our sequence analysis. In summary, these control simulations demonstrate that, while all of the ID-related variants incur substantial changes in the biomechanical characteristics of the OGT-TPR domain, mutations that are not associated with ID phenotypes lead to no, or milder, deviations from the wt properties.

## 6 Conclusion

TPR domains are involved in many key biological processes through their ability to bind selectively to an array of different protein partners. The stacking of repeat units, forming a superhelical global structure, provides TPR domains with high flexibility while retaining a robust protein fold. Here, we have characterised the nanospring character of the OGT-TPR domain and found it to show fully reversible elasticity over a wide range of domain extensions. A small number of single-point mutations within this domain are associated with ID phenotypes. Interestingly, while not all of these mutations lead to changes in the equilibrium domain conformation, they all show strong deviations from the wild-type elasticity and dynamics. Neutral mutations, by contrast, display no or only mild effects. The differences also impact on the energetics of the global conformational changes of the domain that underpin substrate binding and release. Some of these effects may not be detectable in crystal structures, since the average, or dominant, domain conformation often remains unchanged. Taken together, our results suggest that the mutations are likely to display defects upon substrate interaction, due to their altered flexibility and conformational energetics. Our findings may provide a clue towards the ID phenotype of these single-point mutations, which are all distal from the OGT active site but locate to an important part of its substrate binding domain. In addition, they could bring a new perspective to the design of engineered TPR proteins (Alva and Lupas, 2018, Sanchez-deAlcazar et al., 2018) by showing how the dynamics of the domains can be fine-tuned through the introduction of subtle modifications into the repeat sequences.

## 7 Methods

*System Setup.* A shortened construct (residues 22 – 413 from the OGT protein; PDB ID 1W3B) was chosen to model the wt and mutant OGT-TPR domains. This model protein includes repeats TPR2 to TPR11. The constructs were capped and solvated in a triclinic box of 116.6 × 116.6 × 116.6 Å. Na^+^ and Cl^-^ ions were added to neutralise the system and reach a physiological concentration of 0.15 M NaCl. The amber99sb-ildn force field (Lindorff-Larsen et al., 2010) and virtual sites for hydrogen atoms (Feenstra et al., 1999) were used for the protein. The TIP3P water model was used to model the solvent molecules (Jorgensen et al., 1983) and Joung and Cheatham III parameters were used to model the ions (Joung and Cheatham, 2008).

*Unbiased Molecular Dynamics Simulations*. Molecular simulations were performed with the GROMACS molecular dynamics package version 5.1.5 (Abraham et al., 2015). For each system, the geometry was minimized in four cycles that combined 3500 steps of steepest descent algorithm followed by 4500 of conjugate gradient. Thermalization of the system was performed in 10 steps of 2 ns, where the temperature was gradually increased from 50 K to 298 K, while the protein was restrained with a force constant of 10 kJ mol^−1^ Å^−2^. Production runs consisted of four replicates of simulations of 500 ns length for each system (accounting for a total of 2.0 µs of simulation time), using an integration time-step of 4 fs.

The temperature was kept constant by weakly coupling (t = 0.1 ps) the protein and solvent separately to a temperature bath of 298 K with the velocity-rescale thermostat of Bussi et al. (Bussi et al., 2007). The pressure was kept constant at 1 bar using isotropic Berendsen coupling (Berendsen et al., 1984). Long-range electrostatic interactions were calculated using the smooth particle mesh Ewald method (Darden et al., 1993) beyond a short-range Coulomb cut-off of 10 Å. A 10 Å cut-off was also employed for Lennard-Jones interactions. The LINCS algorithm (Hess et al., 1997) was used to restrain the bonds involving hydrogen and SETTLE algorithm (Miyamoto and Kollman, 1992) was used to constrain bond lengths and angles of water molecules. Periodic boundary conditions were applied.

*Analysis of the trajectories and estimation of the spring force constant.* We used MDAnalysis (Gowers et al., 2016, Michaud-Agrawal et al., 2011) and MDtraj (McGibbon et al., 2015) to analyse the trajectories. To estimate the spring force constant, we used the protocol described previously in Kappel et al. (Kappel et al., 2010). Error bars were obtained by bootstrap analysis of the mean of the widths of the Gaussian distributions (1000 cycles).

*TPR domain elastic behaviour.* A slightly different system setup was employed for our Steered Molecular Dynamics simulations (Rief and Grubmüller, 2002). To ensure that any extended state of the proteins fits into the simulation box, proteins were oriented along the z-axis of box vectors. The simulation box was subsequently extended by 60 Å along the z-axis, resulting in a box of 85.5 × 85 × 180 Å. Afterwards, the systems were solvated and a physiological concentration of Na^+^ and Cl^-^ ions was added, replicating the protocols used for the unbiased molecular dynamics simulations. The aforementioned thermalisation and equilibration protocols were also used here. The stretching protocol consisted of fixing the C atoms of the helix TPR1B (N-terminal) using a force constant of 10 kJmol^-1^Å^−2^ and applying a pulling potential of 5 kJmol^-1^Å^−2^ to displace helix TPR12A (C-terminal) at a constant velocity of 1 Å ns^−1^ in the *z*-direction.

## Declaration of Competing Interest

The authors declare that they have no known competing financial interests or personal relationships that could have appeared to influence the work reported in this paper.
