# Supplementary material for: Disease related single point mutations alter the global dynamics of a tetratricopeptide (TPR) α-solenoid domain
Source: J Struct Biol. 2020 Jan 1;209(1):107405. doi: 10.1016/j.jsb.2019.107405 (PMC6961204; doi:10.1016/j.jsb.2019.107405)
Supplement: Supplementary data 1 [file mmc1.docx]

Supporting Information

**Disease related single point mutations alter the global dynamics of a tetratricopeptide (TPR) α-solenoid domain**

Salomé Llabrés^1^, Maxim Tsenkov^1^, Stuart A MacGowan^1^, Geoffrey J Barton^1^, Ulrich Zachariae^1,2^

1 Computational Biology, School of Life Sciences, University of Dundee, Dundee, UK

2 Physics, School of Science and Engineering, University of Dundee, Dundee, UK

Index

Figure S1. Alignment of the OGT-TPR sequence S2

Figure S2. Comparison of the location of ID-related mutations and selected negative control mutations of OGT-TPR sequence S2

Figure S3. Geometrical descriptors of the TPR domains used in this publication. S3

Figure S4. Time evolution of the backbone RMSD of the ID-related variants. S4

Figure S5. Time evolution of the TPR2 – TPR11 distance for the ID-related variants. S4

Figure S6. Time evolution of the backbone RMSD of the negative control mutants. S5

Figure S7. Time evolution of the TPR2 – TPR11 distance for negative control mutants. S5

Figure S8. Geometrical parameters of the wt OGT-TPR domains. S6

Figure S9. Geometrical parameters of the L254F OGT-TPR domains. S7

Figure S10. Geometrical parameters of the A319T OGT-TPR domains. S8

Figure S11. Geometrical parameters of the R284P OGT-TPR domains. S9

Figure S12. Geometrical parameters of the L254I OGT-TPR domains. S10

Figure S13. Geometrical parameters of the A310T OGT-TPR domains. S11

Figure S14. Geometrical parameters of the I279V OGT-TPR domains. S12

Figure S16. Structural details of the mutation sites of the negative control mutants. S13

Figure S17. Distortion caused by the single point mutation L254F. S14

Figure S18. Distortion caused by the single point mutation R284P. S15

Table S1. Prioritisation criteria for selecting control mutations from gnomAD OGT variants S16

Selection of neutral mutations: SMART sequence alignment S17

| 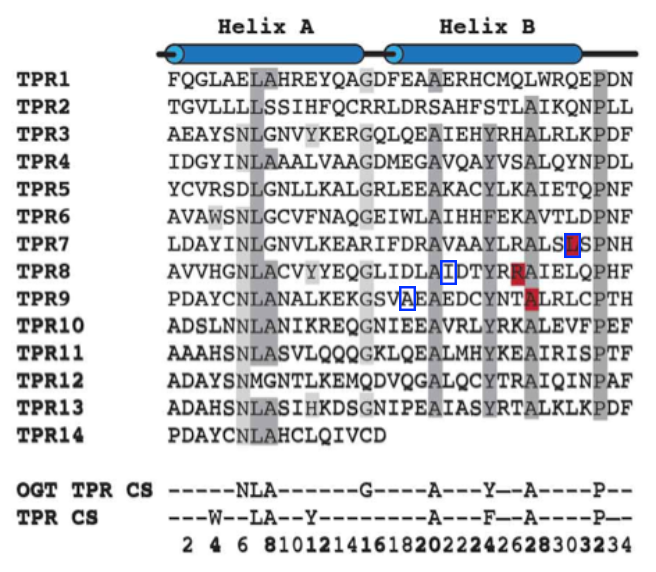 |
| --- |

**Figure S1.** **Alignment of the OGT-TPR sequence**. The ID-related single point mutations are highlighted in red and negative control mutations are highlighted in blue.

| \| **ID-related mutations**  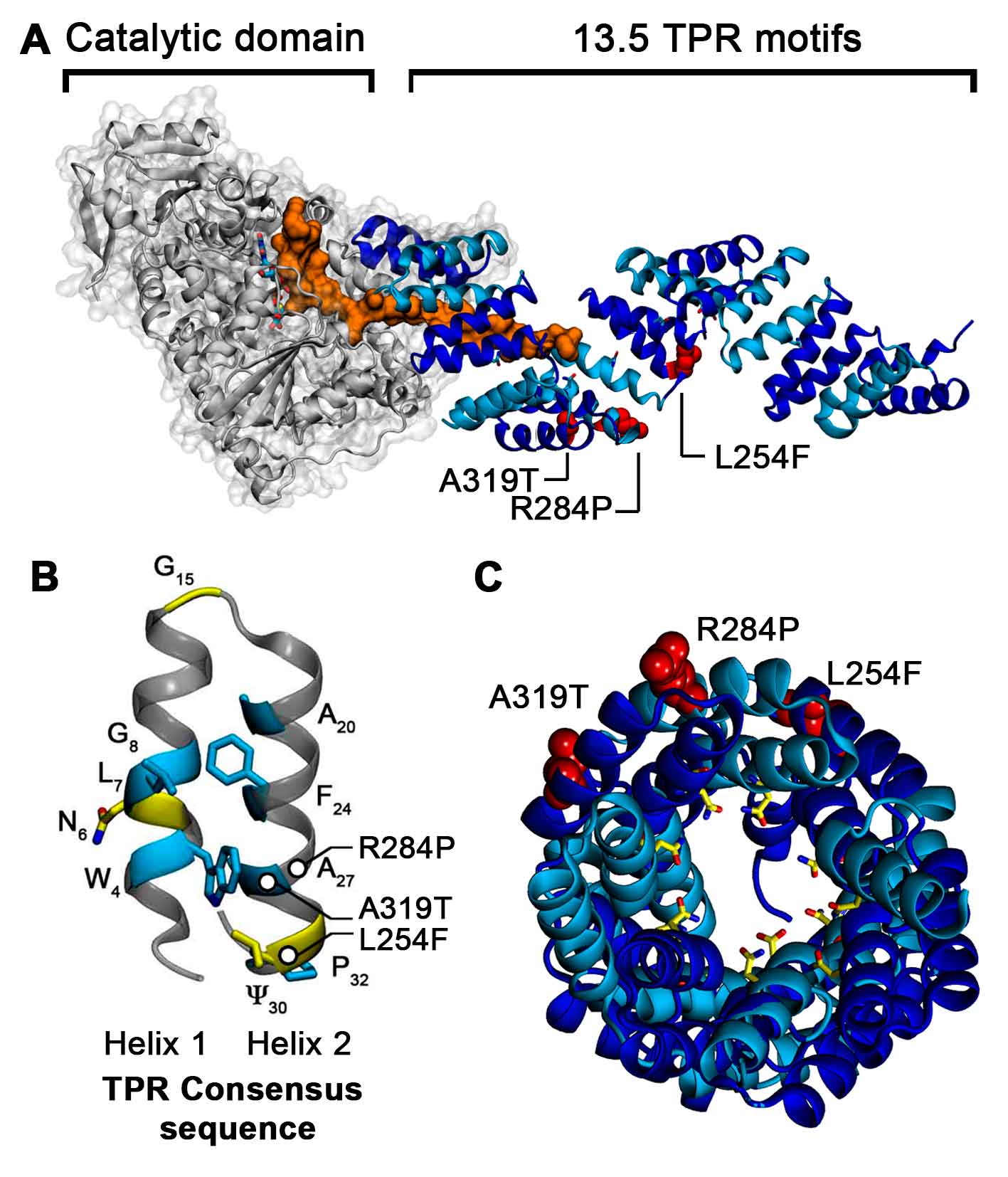 \| **Negative control mutations**  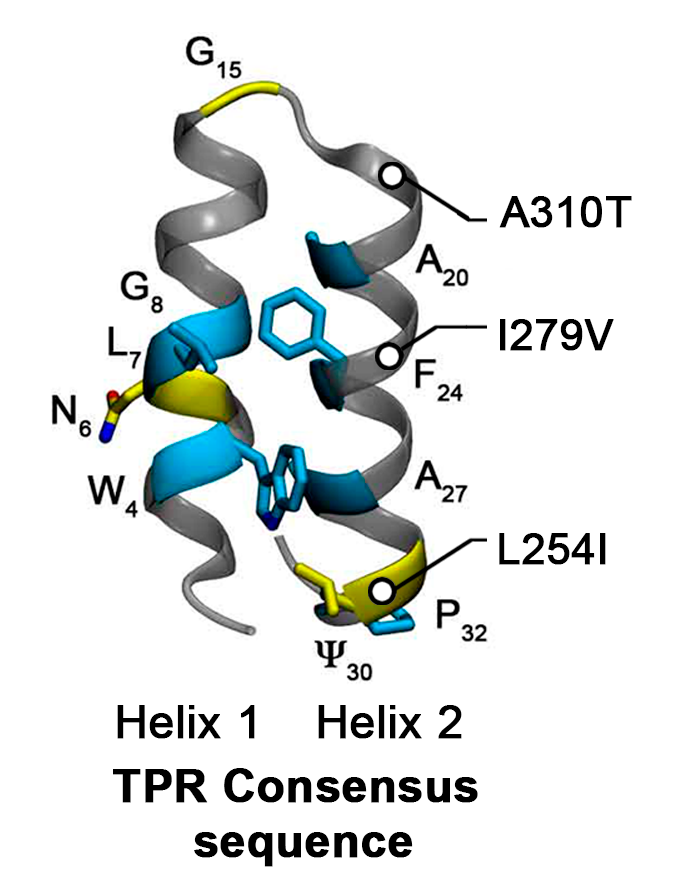 \| \| --- \| --- \| |
| --- | --- | --- |

**Figure S2.** **Comparison of the location of ID-related mutations (**left**) and selected negative control mutations (**right**) of the OGT-TPR sequence**. TPR consensus sequence (CS) is shown as blue sticks and blue cartoon. The additional OGT TPR consensus sequence is shown as yellow sticks.

| 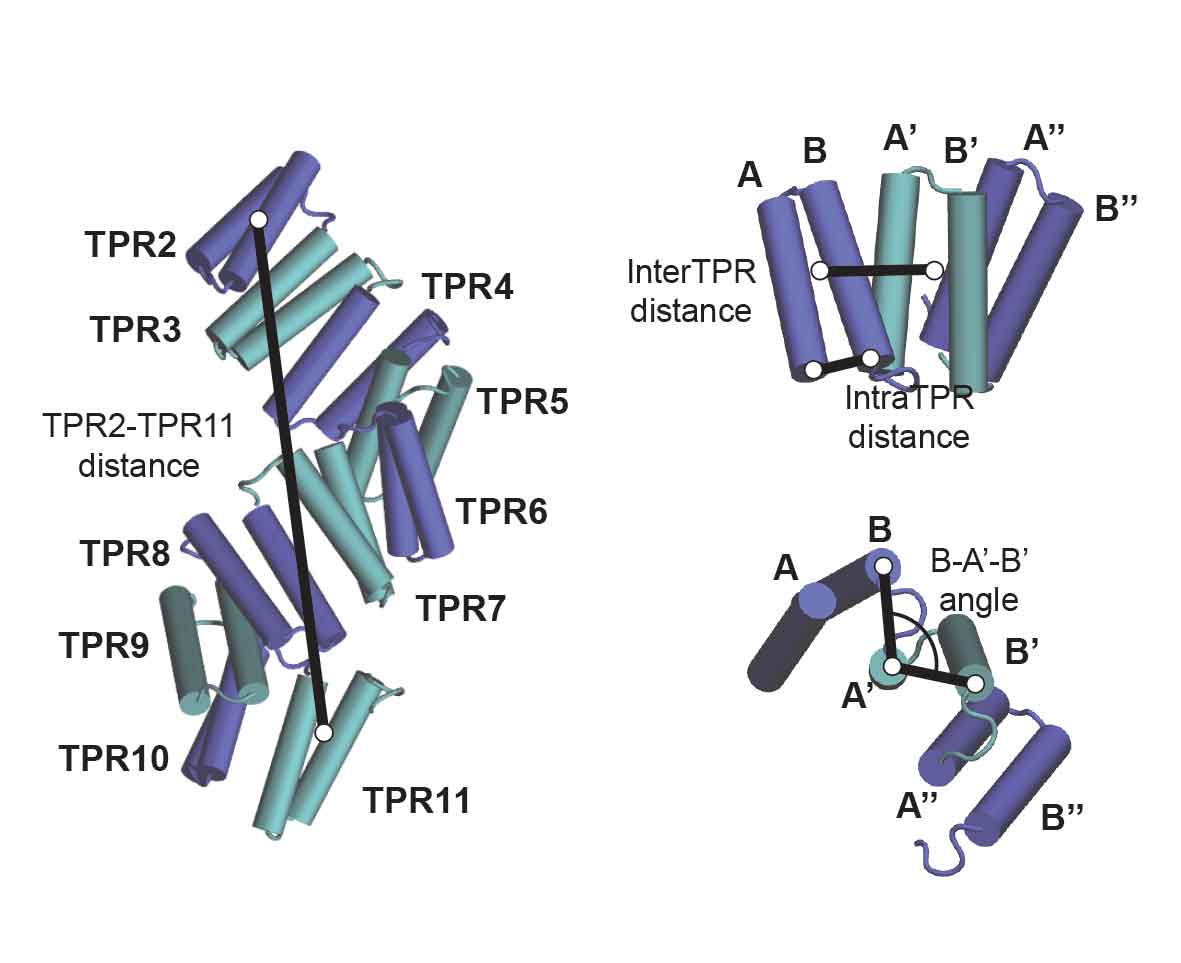 |
| --- |

**Figure S3. Geometrical descriptors of the TPR domains**. Intra-TPR distance is calculated as the distance between the Cα atoms of the positions 0 and 30 of the TPR sequence of each domain. Inter-TPR distance is calculated as the distance between the Centre-Of-Mass (COM) of adjacent TPR domains. The B’-A-B angle is the angle formed by the positions of the positions 0 and 30 of the TPR sequence of each domain and the position 30 of the previous repeat. The TPR2-TPR11 distance is defined by th distance between the COM of the TRP2 and the TPR11 repeats.

| **Wild type**  **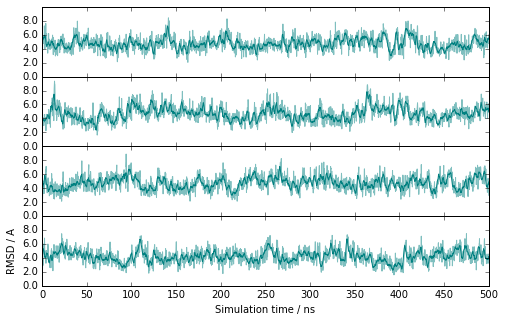** | **L254F mutant**  **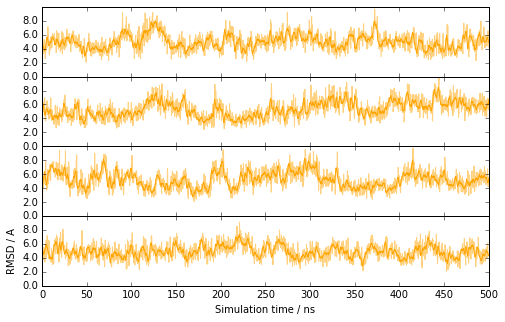** |
| --- | --- |
| **A319T mutant**  **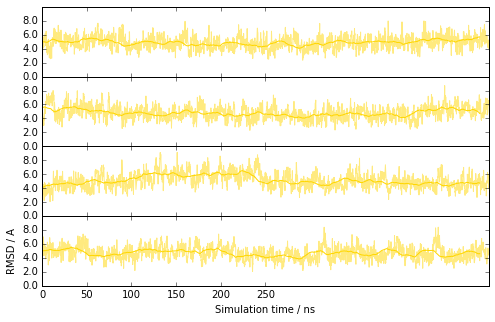** | **R284P mutant**  **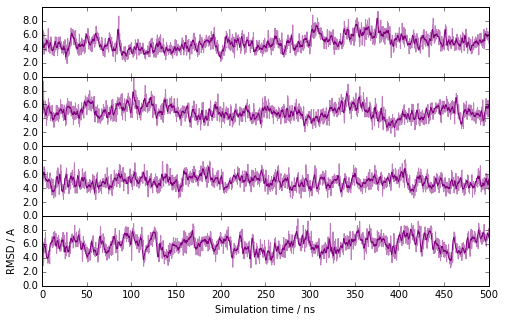** |

**Figure S4. Time evolution of the backbone RMSD of the ID-related variants**. Wild type, L254F, A319T and R284P domains are shown in cyan, orange, yellow and purple plots respectively .

| **Wild type**  **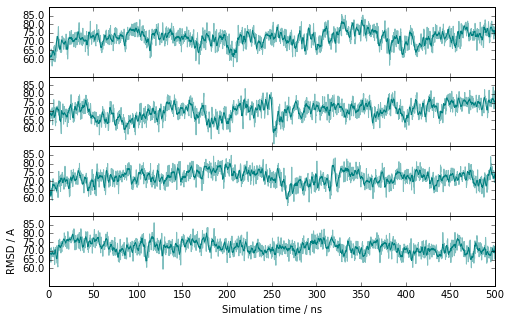** | **L254F mutant**  **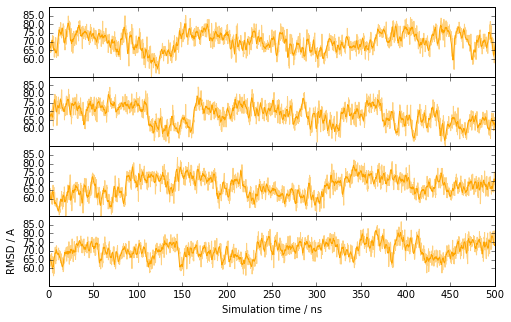** |
| --- | --- |
| **A319T mutant**  **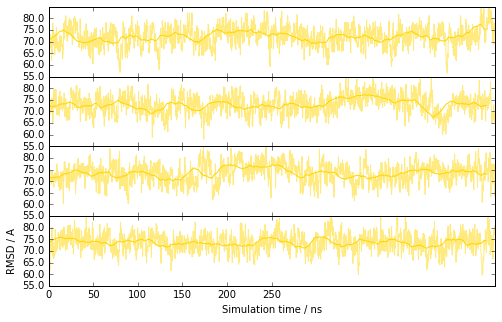** | **R284P mutant**  **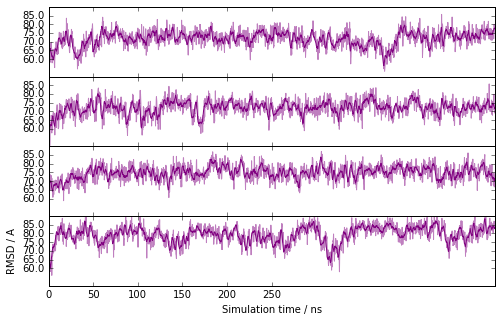** |

**Figure S5. Time evolution of the TPR2 – TPR11 distance for the ID-related mutants.** Wild type, L254F, A319T and R284P domains are shown in cyan, orange, yellow and purple plots respectively.

| **Wild type**  **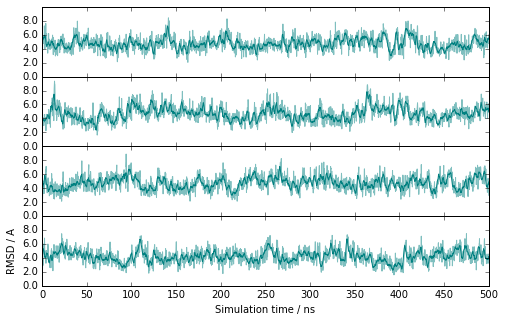** | **L254I mutant**  **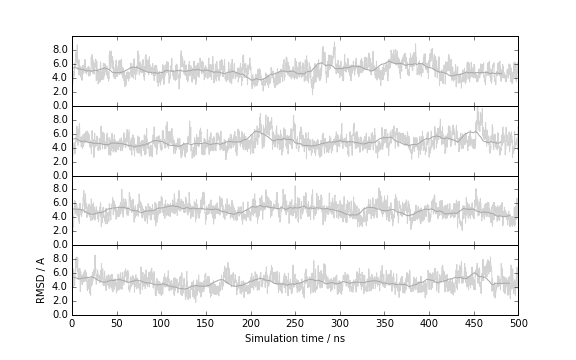** |
| --- | --- |
| **A310T mutant**  **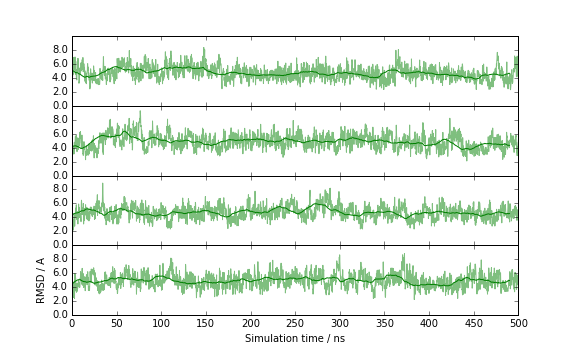** | **I279V mutant**  **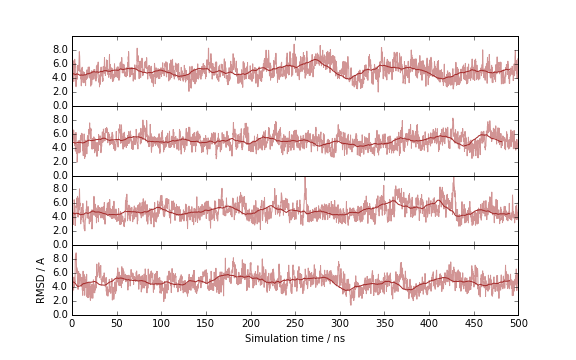** |

**Figure S6. Time evolution of the backbone RMSD of the negative control mutants**. Wild type, L254I, A310T and I279V domains are shown in cyan, orange, yellow and purple plots respectively.

| **Wild type**  **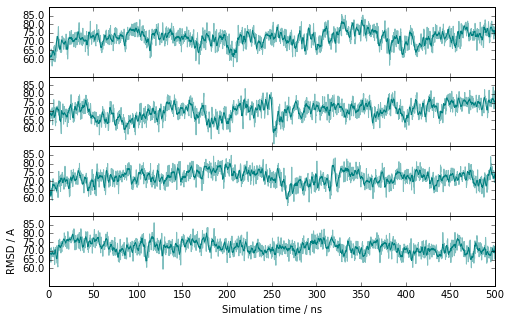** | **L254I mutant**  **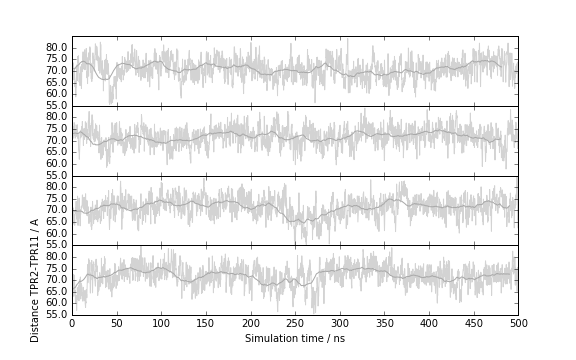** |
| --- | --- |
| **A310T mutant**  **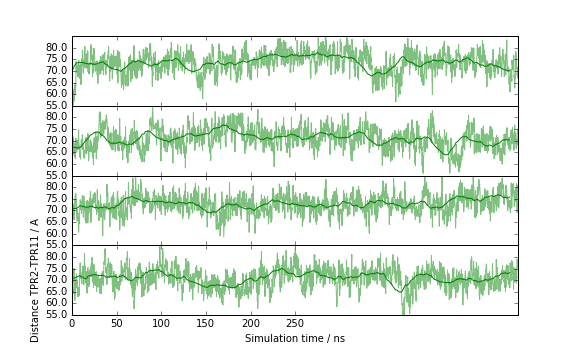** | **I279V mutant**  **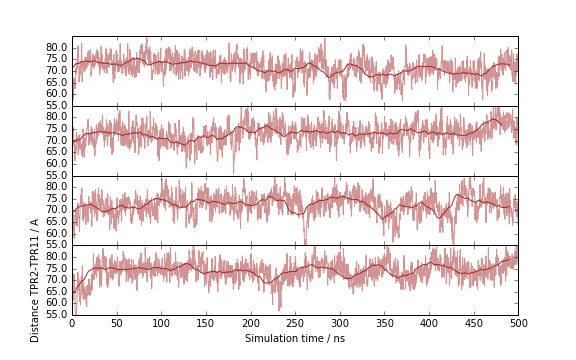** |

**Figure S7. Time evolution of the TPR2 – TPR11 distance for the negative control mutants.** Wild type, L254I, A310T and I279V domains are shown in cyan, orange, yellow and purple plots respectively.

| **IntraTPR Distance**  **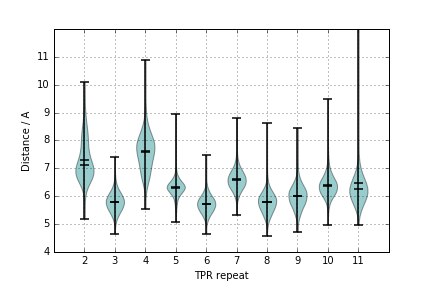** |
| --- |
| **InterTPR Distance**  **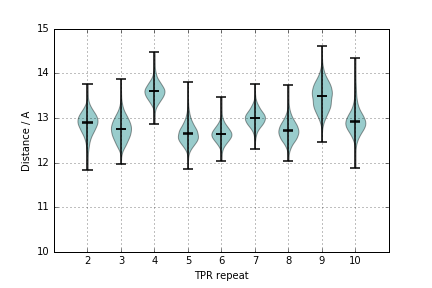** |
| **B-A’-B’ Angle**  **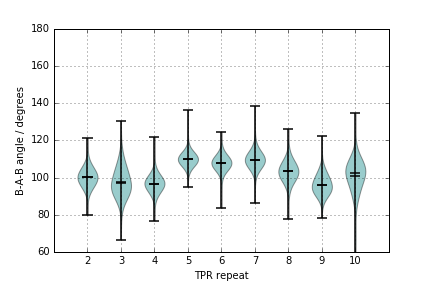** |

**Figure S8. Geometrical parameters of the *wt* OGT-TPR domains.** Violin plots highlight the distributions of each repeat and the mean and the median values.

| **IntraTPR Distance**  **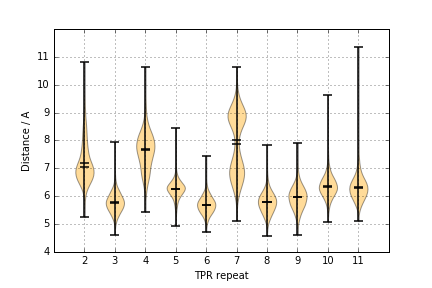** |
| --- |
| **InterTPR Distance**  **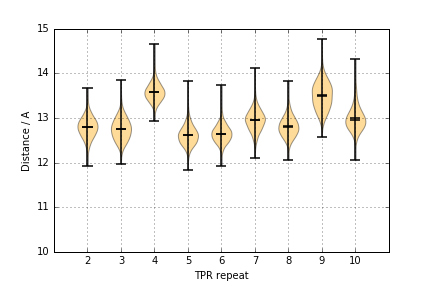** |
| **B-A’-B’ Angle**  **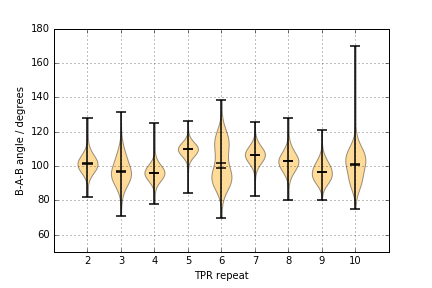** |

**Figure S9. Geometrical parameters of the *L254F* OGT-TPR domains.** Violin plots highlight the distributions of each repeat and the mean and the median values.

| **IntraTPR Distance**  **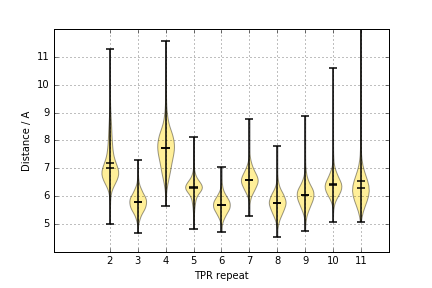** |
| --- |
| **InterTPR Distance**  **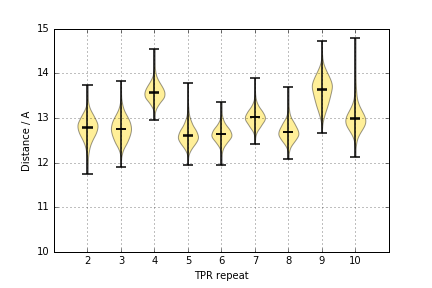** |
| **B-A’-B’ Angle**  **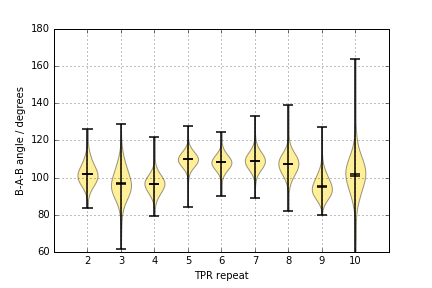** |

**Figure S10. Geometrical parameters of the *A319T* OGT-TPR domains.** Violin plots highlight the distributions of each repeat and the mean and the median values.

| **IntraTPR Distance**  **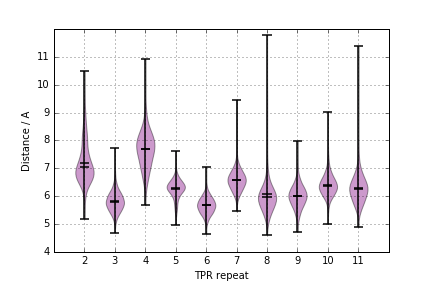** |
| --- |
| **InterTPR Distance**  **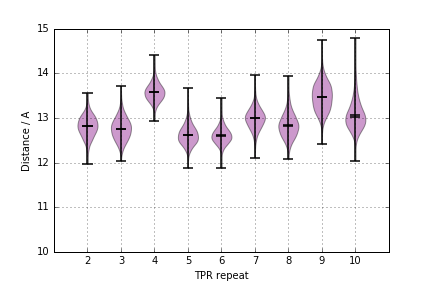** |
| **B-A’-B’ Angle**  **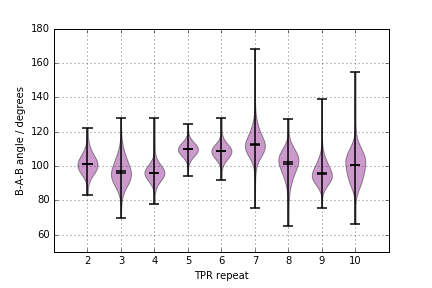** |

**Figure S11. Geometrical parameters of the *R284P* OGT-TPR domains**. Violin plots highlight the distributions of each repeat and the mean and the median values.

| **IntraTPR Distance**  **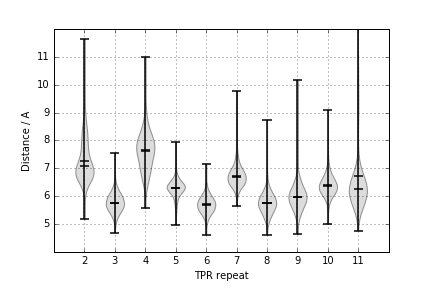** |
| --- |
| **InterTPR Distance**  **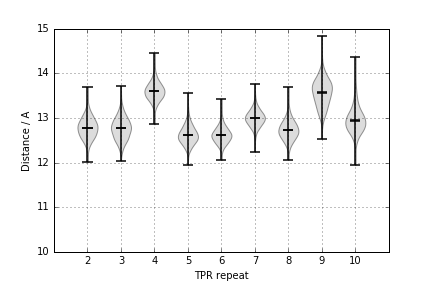** |
| **B-A’-B’ Angle**  **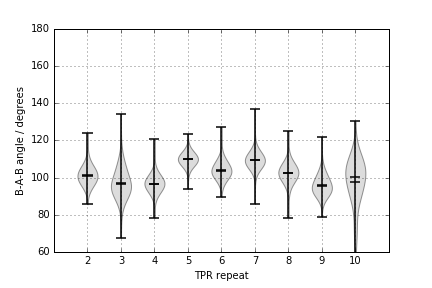** |

**Figure S12. Geometrical parameters of the *L254I* OGT-TPR domains**. Violin plots highlight the distributions of each repeat and the mean and the median values.

| **IntraTPR Distance**  **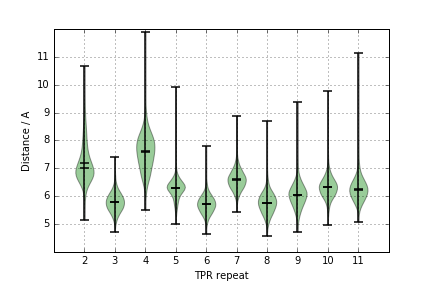** |
| --- |
| **InterTPR Distance**  **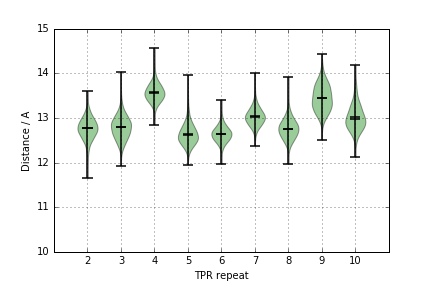** |
| **B-A’-B’ Angle**  **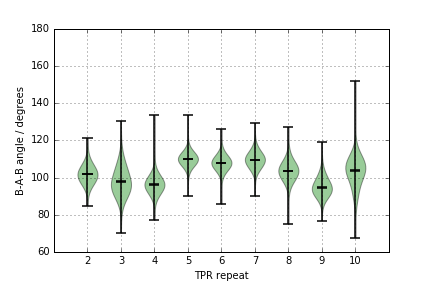** |

**Figure S13. Geometrical parameters of the *A310T* OGT-TPR domains**. Violin plots highlight the distributions of each repeat and the mean and the median values.

| **IntraTPR Distance**  **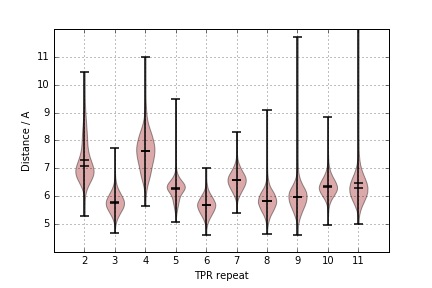** |
| --- |
| **InterTPR Distance**  **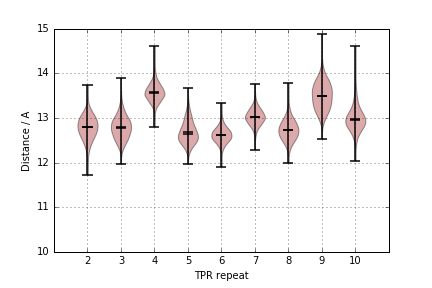** |
| **B-A’-B’ Angle**  **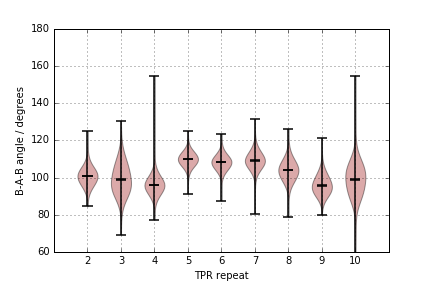** |

**Figure S14. Geometrical parameters of the *I279V* OGT-TPR domains**. Violin plots highlight the distributions of each repeat and the mean and the median values.


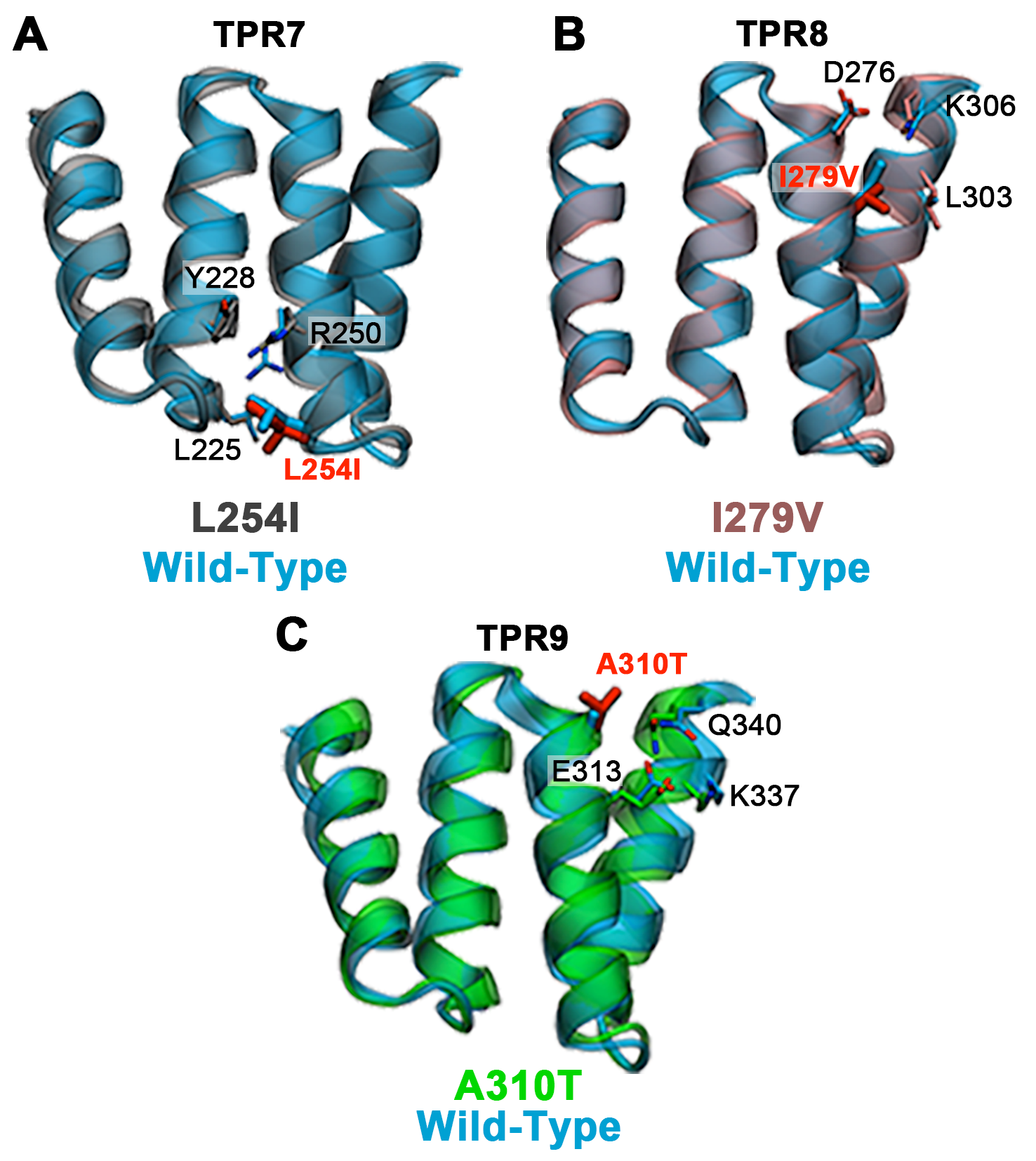


**Figure S15.** **Structural details of the mutation sites of the negative control mutants. A)** Comparison between wildtype and L254I OGT-TPR domains. **B)** Comparison between wildtype and I279V OGT-TPR domains. **C)** Comparison between wildtype and A310T OGT-TPR domains. Wildtype, L254I, I279V and A310T TPR domains are shown as blue, grey, pink and green cartoon respectively. Mutations are highlighted in red sticks.

| **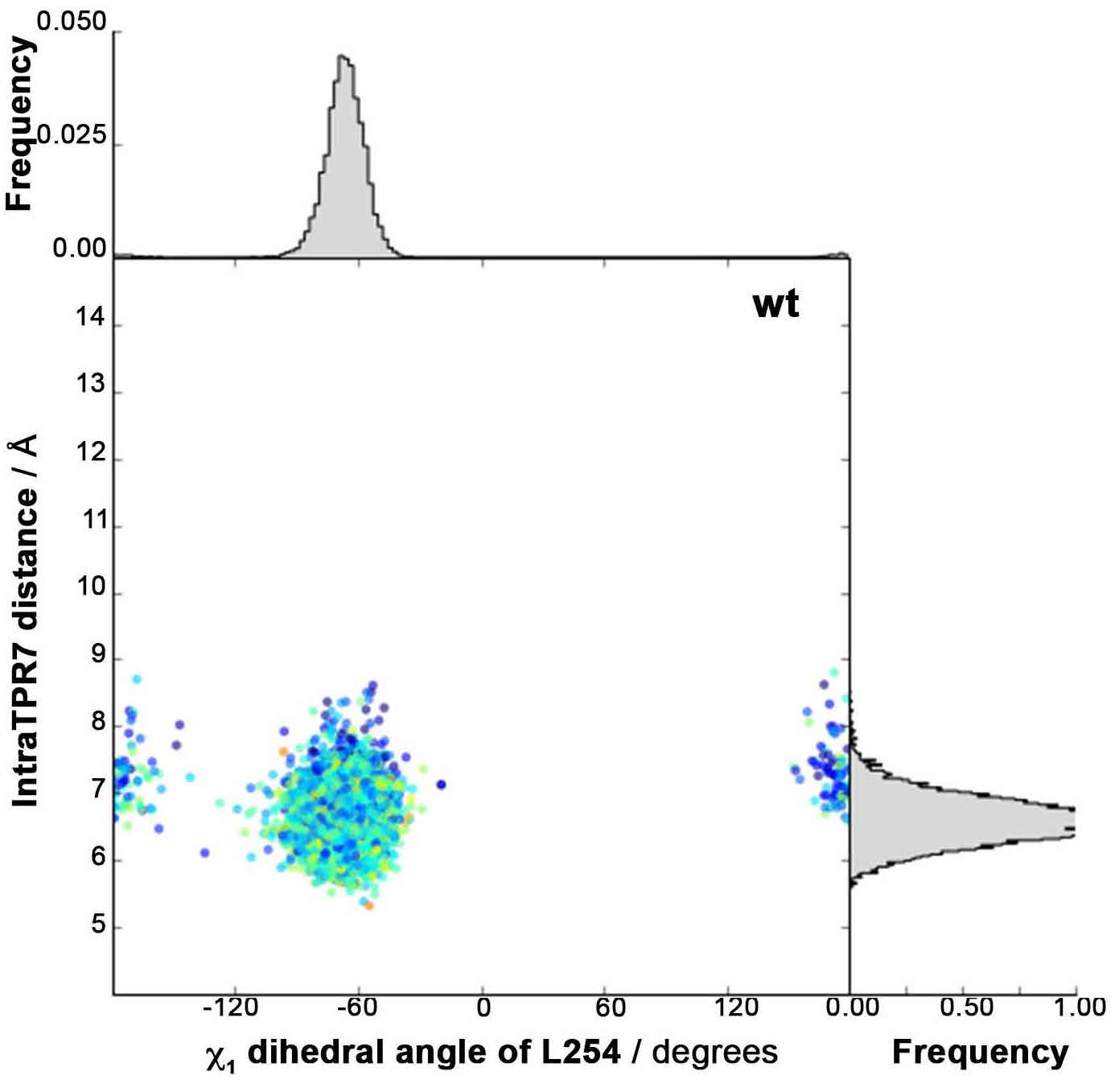** |
| --- |
| 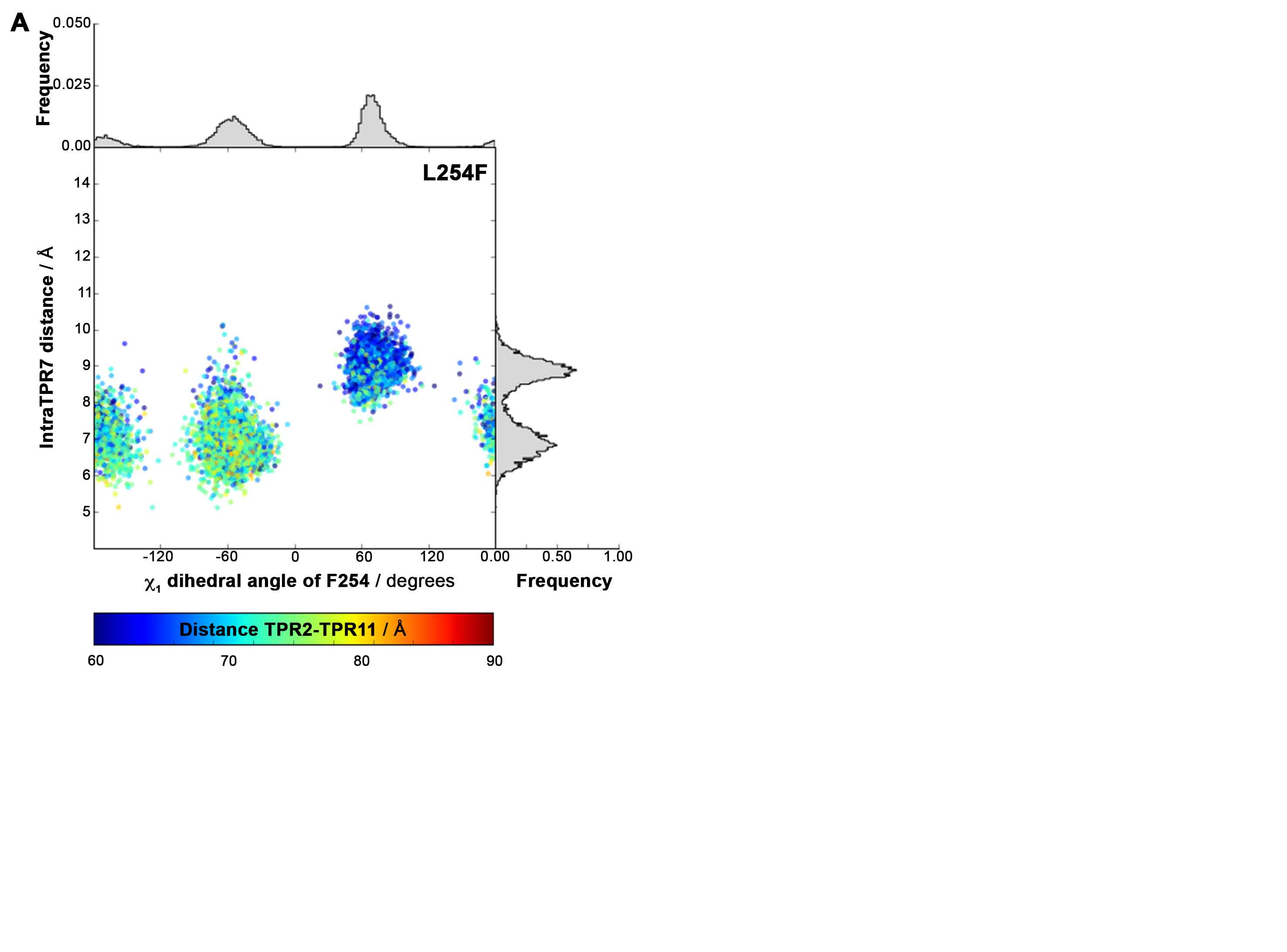 |

**Figure S16.** Distortion caused by the single point mutation L254F on the X1 dihedral angle of the residue 254, the intraTPR7 distance and TPR2-TPR11 distance.

| **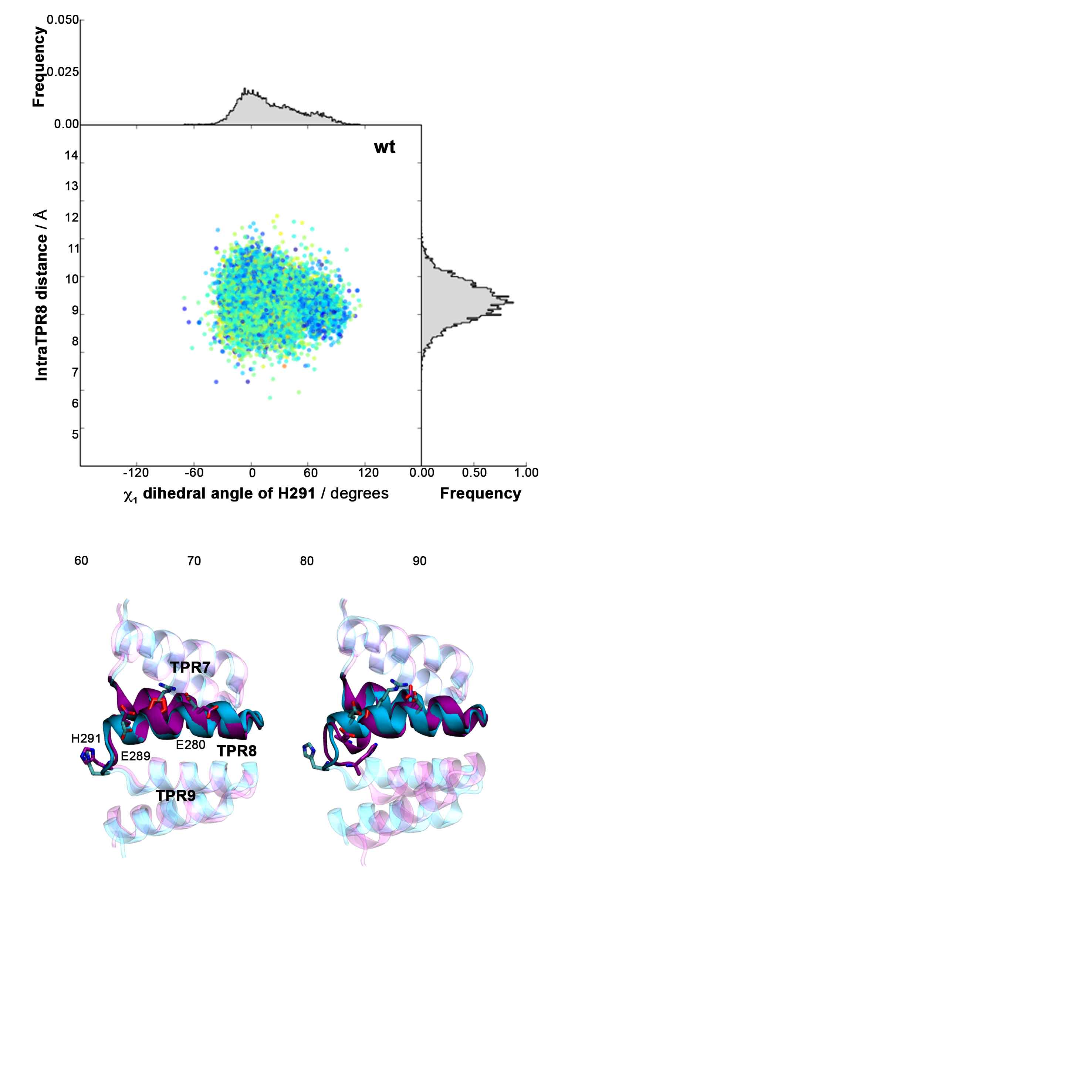** |
| --- |
| 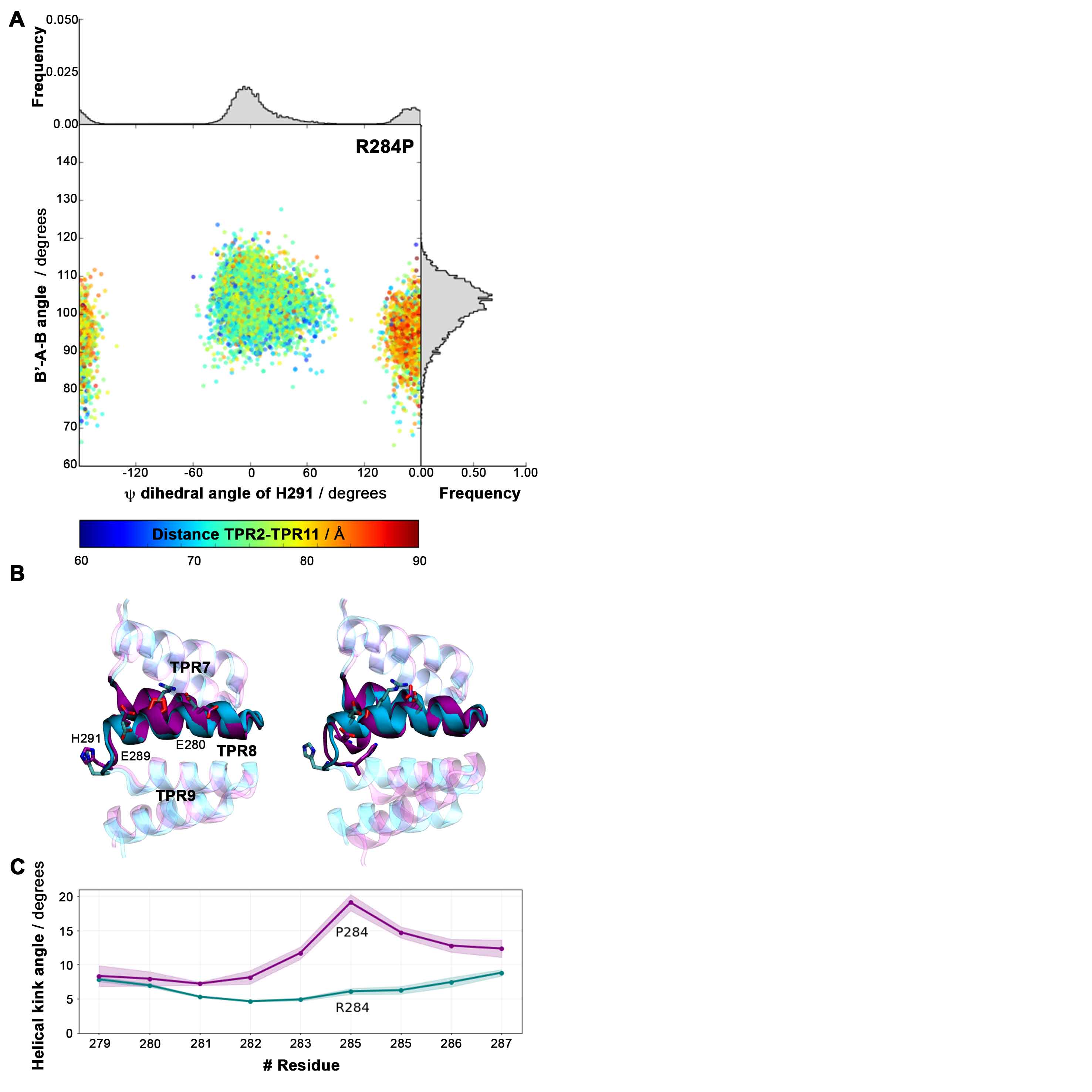 |

**Figure S17.** Distortion caused by the single point mutation R284P on the X1 dihedral angle of the residue 291, the B’-A-B angle between TPR98 and TPR9 and TPR2-TPR11 distance.

**Table S1:** Prioritisation criteria for selecting control mutations from gnomAD OGT variants

| **TPR Residue Positions** | **TPR Number** | **Variant** | **Substitution (Zvelebil)^1^** | **Consensus Position^2^** | **Consensus Sequence^2^** | **Allele**  **Count (Male)** | **Allele**  **Count (Female)** |
| --- | --- | --- | --- | --- | --- | --- | --- |
| 259-292 | 8 | I279V | 0.9 | 21 | L | 2 | 12 |
| 293-326 | 9 | A310T | 0.8 | 18 | E | 1 | 1 |
| 123-156 | 4 | A136V | 0.8 | 14 | L | 2 | 1 |
| 327-360 | 10 | I343T | 0.7 | 17 | Y | 2 | 1 |
| 55-88 | 2 | L87V | 0.9 | 33 | D | 2 | 1 |
| 89-122 | 3 | R117H | 0.8 | 29 | E | 2 | 0 |
| 89-122 | 3 | I109V | 0.9 | 21 | L | 1 | 0 |
| 89-122 | 3 | I109T | 0.7 | 21 | L | 1 | 0 |
| 89-122 | 3 | P120S | 0.7 | 32 | P | 1 | 0 |
| 191-224 | 6 | N196K | 0.5 | 6 | N | 0 | 2 |
| 259-292 | 8 | D276N | 0.9 | 18 | E | 0 | 2 |
| 361-394 | 11 | R389Q | 0.8 | 29 | E | 0 | 2 |
| 123-156 | 4 | M139V | 0.8 | 17 | Y | 0 | 1 |
| 123-156 | 4 | V147I | 0.9 | 25 | E | 0 | 1 |
| 157-190 | 5 | T186M | 0.8 | 30 | L | 0 | 1 |
| 327-360 | 10 | N332T | 0.8 | 6 | N | 0 | 1 |
| 327-360 | 10 | K337T | 0.7 | 11 | Y | 0 | 1 |
| 327-360 | 10 | V347F | 0.7 | 21 | L | 0 | 1 |
| 429-462 | 13 | R453G | 0.4 | 25 | E | 0 | 1 |
| 55-88 | 2 | N85K | 0.5 | 31 | D | 0 | 1 |
| 89-122 | 3 | R113Q | 0.8 | 25 | E | 0 | 1 |
| 89-122 | 3 | R117C | 0.5 | 29 | E | 0 | 1 |

^1^ A measure of the conservation of amino acid properties (Zvelebil et al., 1987).

^2^ Consensus positions refer to an alignment of human TPR annotations of length 34 in the SMART database obtained via InterPro (Letunic and Bork, 2017; Mitchell et al., 2019) (see Supplementary SMART sequence alignment, pp. S17-29).

**Supplementary SMART sequence alignment**

>sp|A0AVF1|IFT56_HUMAN/91-124

SEVWVNLACTYFFLGMYKQAEAAGFKASKSRLQN

>sp|A0AVF1|IFT56_HUMAN/151-184

TEDQLSLASIHYMRSHYQEAIDIYKRILLDNREY

>sp|A0AVF1|IFT56_HUMAN/57-90

EDTNLWIGYCAFHLGDYKRALEEYENATKEENCN

>sp|A0AVF1|IFT56_HUMAN/461-494

FSLLQLIANDCYKMGQFYYSAKAFDVLERLDPNP

>sp|A0AVF1|IFT56_HUMAN/230-263

FRLYNGRAAEAELKSLMDNASSSFEFAKELIRHN

>sp|A2A3L6|TTC24_HUMAN/236-269

GHLYNDLGLGYSQLQLFPLAVEAFLQALPLCWVP

>sp|A2A3L6|TTC24_HUMAN/117-150

GDQCFNVALAYHALGELPQALAWYHRALGHYQPQ

>sp|A2A3L6|TTC24_HUMAN/154-187

GEAWAKMGACYQALGQPELAAHCLQEASQAYAQE

>sp|A2A3L6|TTC24_HUMAN/36-69

IQALTRAGHGALQAGQNHEALNNFQRAFLLASKA

>sp|A2A3L6|TTC24_HUMAN/313-346

GRSFGSLAFALSQLGDHKAARDNYLHALQAARDS

>sp|A2A3L6|TTC24_HUMAN/353-386

WQACEGLGAAAARLGQYDQALKYYKEALAQCQKE

>sp|A2A3L6|TTC24_HUMAN/273-306

ATVLRNLGMAHNALGNYQEAREFHQKAADLHGSV

>sp|A6NKT7|RGPD3_HUMAN/60-93

PRAHRFLGLLYELEENTEKAVECYRRSVELNPTQ

>sp|A6NLP5|TTC36_HUMAN/85-118

ASAYNNRAQARRLQGDVAGALEDLERAVELSGGR

>sp|A6NLP5|TTC36_HUMAN/51-84

SKALELQGVMAAEAGDLSTALERFGQAICLLPER

>sp|A6NLP5|TTC36_HUMAN/123-156

RQSFVQRGLLARLQGRDDDARRDFERAARLGSPF

>sp|A8MYJ7|TTC34_HUMAN/464-497

AEDFCRQGRLLLSLGDEAAAAGAFAQALKLAPSL

>sp|A8MYJ7|TTC34_HUMAN/424-457

ACHLRLRATCLAELQEFGRALRDLDHVLQEALGD

>sp|A8MYJ7|TTC34_HUMAN/212-245

VQALCGRALVHLALDQLQEAVDDIVSALKLGPGT

>sp|A8MYJ7|TTC34_HUMAN/306-339

PHWHLLLADILMAQGSYEEAGTHLEKALHRAPTS

>sp|A8MYJ7|TTC34_HUMAN/340-373

EAARARLGLLQLKKGDVPGAARDLQSLAEVDAPD

>sp|A8MYJ7|TTC34_HUMAN/512-545

ARMFLLRGQCCLEEQRHAEAWTAVESGLLVDPDH

>sp|A8MYJ7|TTC34_HUMAN/178-211

SESLLARARCYGFLGQKKTAMFDFNTVLRAEPGN

>sp|O14607|UTY_HUMAN/315-348

ADTWCSIGVLYQQQNQPMDALQAYICAVQLDHGH

>sp|O14607|UTY_HUMAN/281-314

GQSWYFLGRCYSSIGKVQDAFISYRQSIDKSEAS

>sp|O14607|UTY_HUMAN/202-235

AEIQFHIAHLYETQRKYHSAKEAYEQLLQTENLP

>sp|O14607|UTY_HUMAN/127-160

AAFLYGLGLVYFYYNAFHWAIKAFQDVLYVDPSF

>sp|O14607|UTY_HUMAN/349-382

AAAWMDLGTLYESCNQPQDAIKCYLNAARSKRCS

>sp|O14607|UTY_HUMAN/90-123

SDFFCQLGHFNLLLEDYSKALSAYQRYYSLQADY

>sp|O14715|RGPD8_HUMAN/60-93

PKAHRFLGLLYELEENTEKAVECYRRSVELNPTQ

>sp|O14879|IFIT3_HUMAN/94-127

LVTWGNYAWVYYHLGRLSDAQIYVDKVKQTCKKF

>sp|O14879|IFIT3_HUMAN/51-84

ATMYNLLAYIKHLDGNNEAALECLRQAEELIQQE

>sp|O14879|IFIT3_HUMAN/136-169

SELDCEEGWTQLKCGRNERAKVCFEKALEEKPNN

>sp|O14879|IFIT3_HUMAN/415-448

PNYWYLQGLIHKQNGDLLQAAKCYEKELGRLLRD

>sp|O14879|IFIT3_HUMAN/241-274

TDVLRSAAKFYRRKGDLDKAIELFQRVLESTPNN

>sp|O15050|TRNK1_HUMAN/15-48

AVLLCNKSNAFFSLGKWNEAFVAAKECLQWDPTY

>sp|O15050|TRNK1_HUMAN/49-82

VKGYYRAGYSLLRLHQPYEAARMFFEGLRLVQRS

>sp|O15287|FANCG_HUMAN/394-427

PEVFLEAAVALIQAGRAQDALTLCEELLSRTSSL

>sp|O15287|FANCG_HUMAN/514-547

AAALISRGLEWVASGQDTKALQDFLLSVQMCPGN

>sp|O15287|FANCG_HUMAN/246-279

VQVYTALGSCHRKMGNPQRALLYLVAALKEGSAW

>sp|O15294|OGT1_HUMAN/395-428

ADAYSNMGNTLKEMQDVQGALQCYTRAIQINPAF

>sp|O15294|OGT1_HUMAN/259-292

AVVHGNLACVYYEQGLIDLAIDTYRRAIELQPHF

>sp|O15294|OGT1_HUMAN/327-360

ADSLNNLANIKREQGNIEEAVRLYRKALEVFPEF

>sp|O15294|OGT1_HUMAN/89-122

AEAYSNLGNVYKERGQLQEAIEHYRHALRLKPDF

>sp|O15294|OGT1_HUMAN/225-258

LDAYINLGNVLKEARIFDRAVAAYLRALSLSPNH

>sp|O15294|OGT1_HUMAN/361-394

AAAHSNLASVLQQQGKLQEALMHYKEAIRISPTF

>sp|O15294|OGT1_HUMAN/429-462

ADAHSNLASIHKDSGNIPEAIASYRTALKLKPDF

>sp|O15294|OGT1_HUMAN/157-190

YCVRSDLGNLLKALGRLEEAKACYLKAIETQPNF

>sp|O15294|OGT1_HUMAN/123-156

IDGYINLAAALVAAGDMEGAVQAYVSALQYNPDL

>sp|O15294|OGT1_HUMAN/21-54

FQGLAELAHREYQAGDFEAAERHCMQLWRQEPDN

>sp|O15294|OGT1_HUMAN/191-224

AVAWSNLGCVFNAQGEIWLAIHHFEKAVTLDPNF

>sp|O15294|OGT1_HUMAN/293-326

PDAYCNLANALKEKGSVAEAEDCYNTALRLCPTH

>sp|O15550|KDM6A_HUMAN/130-163

AAFLYGLGLVYFHYNAFQWAIKAFQEVLYVDPSF

>sp|O15550|KDM6A_HUMAN/318-351

ADTWCSIGVLYQQQNQPMDALQAYICAVQLDHGH

>sp|O15550|KDM6A_HUMAN/205-238

AEIQFHIAHLYETQRKYHSAKEAYEQLLQTENLS

>sp|O15550|KDM6A_HUMAN/93-126

SDFFCQLGHFNLLLEDYPKALSAYQRYYSLQSDY

>sp|O15550|KDM6A_HUMAN/284-317

GQSWYFLGRCYSSIGKVQDAFISYRQSIDKSEAS

>sp|O15550|KDM6A_HUMAN/352-385

AAAWMDLGTLYESCNQPQDAIKCYLNATRSKSCS

>sp|O43765|SGTA_HUMAN/91-124

AERLKTEGNEQMKVENFEAAVHFYGKAIELNPAN

>sp|O43765|SGTA_HUMAN/159-192

SKAYGRMGLALSSLNKHVEAVAYYKKALELDPDN

>sp|O43765|SGTA_HUMAN/125-158

AVYFCNRAAAYSKLGNYAGAVQDCERAICIDPAY

>sp|O75344|FKBP6_HUMAN/219-252

LPVLLNLSFTYLKLDRPTIALCYGEQALIIDQKN

>sp|O75344|FKBP6_HUMAN/171-204

AATEREFGNYLFRQNRFYDAKVRYKRALLLLRRR

>sp|O75344|FKBP6_HUMAN/253-286

AKALFRCGQACLLLTEYQKARDFLVRAQKEQPFN

>sp|O76094|SRP72_HUMAN/406-439

PGMVSALVTMYSHEEDIDSAIEVFTQAIQWYQNH

>sp|O76094|SRP72_HUMAN/11-44

VPALWSEVNRYGQNGDFTRALKTVNKILQINKDD

>sp|O76094|SRP72_HUMAN/226-259

AIIHGQMAYILQLQGRTEEALQLYNQIIKLKPTD

>sp|O76094|SRP72_HUMAN/447-480

LSLIREAANFKLKYGRKKEAISDLQQLWKQNPKD

>sp|O76094|SRP72_HUMAN/176-209

HELCYNTACALIGQGQLNQAMKILQKAEDLCRRS

>sp|O94826|TOM70_HUMAN/367-400

ANALIKRGSMYMQQQQPLLSTQDFNMAADIDPQN

>sp|O94826|TOM70_HUMAN/545-578

DFAYETMGTIEVQRGNMEKAIDMFNKAINLAKSE

>sp|O94826|TOM70_HUMAN/401-434

ADVYHHRGQLKILLDQVEEAVADFDECIRLRPES

>sp|O94826|TOM70_HUMAN/442-475

CFALYRQAYTGNNSSQIQAAMKGFEEVIKKFPRC

>sp|O94826|TOM70_HUMAN/329-362

AEALLLRATFYLLIGNANAAKPDLDKVISLKEAN

>sp|O94826|TOM70_HUMAN/187-220

VKALFRRAKAHEKLDNKKECLEDVTAVCILEGFQ

>sp|O94826|TOM70_HUMAN/153-186

STFYQNRAAAFEQLQKWKEVAQDCTKAVELNPKY

>sp|O94826|TOM70_HUMAN/114-147

AQAAKNKGNKYFKAGKYEQAIQCYTEAISLCPTE

>sp|O94826|TOM70_HUMAN/476-509

AEGYALYAQALTDQQQFGKADEMYDKCIDLEPDN

>sp|O94906|PRP6_HUMAN/709-742

PKLWMMKGQIEEQKEMMEKAREAYNQGLKKCPHS

>sp|O94906|PRP6_HUMAN/743-776

TPLWLLLSRLEEKIGQLTRARAILEKSRLKNPKN

>sp|O94906|PRP6_HUMAN/841-874

PHVLLAVAKLFWSQRKITKAREWFHRTVKIDSDL

>sp|O95801|TTC4_HUMAN/117-150

AVLYTNRAAAQYYLGNFRSALNDVTAARKLKPCH

>sp|O95801|TTC4_HUMAN/151-184

LKAIIRGALCHLELKHFAEAVNWCDEGLQIDAKE

>sp|O95801|TTC4_HUMAN/79-112

AKTYKDEGNDYFKEKDYKKAVISYTEGLKKKCAD

>sp|P09913|IFIT2_HUMAN/247-280

TDVLRSAAKFYRRKDEPDKAIELLKKALEYIPNN

>sp|P09913|IFIT2_HUMAN/51-84

ATMCNLLAYLKHLKGQNEAALECLRKAEELIQQE

>sp|P09914|IFIT1_HUMAN/95-128

LVTWGNFAWMYYHMGRLAEAQTYLDKVENICKKL

>sp|P09914|IFIT1_HUMAN/141-174

IDCEEGWALLKCGGKNYERAKACFEKVLEVDPEN

>sp|P09914|IFIT1_HUMAN/251-284

TYVFRYAAKFYRRKGSVDKALELLKKALQETPTS

>sp|P09914|IFIT1_HUMAN/52-85

VGIHNLLAYVKHLKGQNEEALKSLKEAENLMQEE

>sp|P09914|IFIT1_HUMAN/437-470

LESLSLLGFVYKLEGNMNEALEYYERALRLAADF

>sp|P09914|IFIT1_HUMAN/340-373

EVAHLDLARMYIEAGNHRKAEENFQKLLCMKPVV

>sp|P0DJD0|RGPD1_HUMAN/51-84

PRAHRFLGLLYELEENTEKAVECYRRSLELNPPQ

>sp|P0DJD1|RGPD2_HUMAN/59-92

PRAHRFLGLLYELEENTEKAVECYRRSLELNPPQ

>sp|P19878|NCF2_HUMAN/71-104

AVAYFQRGMLYYQTEKYDLAIKDLKEALIQLRGN

>sp|P19878|NCF2_HUMAN/121-154

CEVLYNIAFMYAKKEEWKKAEEQLALATSMKSEP

>sp|P19878|NCF2_HUMAN/37-70

SRICFNIGCMYTILKNMTEAEKAFTRSINRDKHL

>sp|P30260|CDC27_HUMAN/499-532

GWVLCQIGRAYFELSEYMQAERIFSEVRRIENYR

>sp|P30260|CDC27_HUMAN/635-668

YNAWYGLGMIYYKQEKFSLAEMHFQKALDINPQS

>sp|P30260|CDC27_HUMAN/567-600

PEAWCAAGNCFSLQREHDIAIKFFQRAIQVDPNY

>sp|P30260|CDC27_HUMAN/737-770

SLVYFLIGKVYKKLGQTHLALMNFSWAMDLDPKG

>sp|P30260|CDC27_HUMAN/669-702

SVLLCHIGVVQHALKKSEKALDTLNKAIVIDPKN

>sp|P30260|CDC27_HUMAN/703-736

PLCKFHRASVLFANEKYKSALQELEELKQIVPKE

>sp|P30260|CDC27_HUMAN/601-634

AYAYTLLGHEFVLTEELDKALACFRNAIRVNPRH

>sp|P30260|CDC27_HUMAN/115-148

CFTLSLLGHVYCKTDRLAKGSECYQKSLSLNPFL

>sp|P31948|STIP1_HUMAN/259-292

MTYITNQAAVYFEKGDYNKCRELCEKAIEVGREN

>sp|P31948|STIP1_HUMAN/428-461

IKGYTRKAAALEAMKDYTKAMDVYQKALDLDSSC

>sp|P31948|STIP1_HUMAN/300-333

AKAYARIGNSYFKEEKYKDAIHFYNKSLAEHRTP

>sp|P31948|STIP1_HUMAN/225-258

ALKEKELGNDAYKKKDFDTALKHYDKAKELDPTN

>sp|P31948|STIP1_HUMAN/4-37

VNELKEKGNKALSVGNIDDALQCYSEAIKLDPHN

>sp|P31948|STIP1_HUMAN/360-393

ALEEKNKGNECFQKGDYPQAMKHYTEAIKRNPKD

>sp|P31948|STIP1_HUMAN/394-427

AKLYSNRAACYTKLLEFQLALKDCEECIQLEPTF

>sp|P31948|STIP1_HUMAN/38-71

HVLYSNRSAAYAKKGDYQKAYEDGCKTVDLKPDW

>sp|P31948|STIP1_HUMAN/72-105

GKGYSRKAAALEFLNRFEEAKRTYEEGLKHEANN

>sp|P49321|NASP_HUMAN/43-76

AKKLLGLGQKHLVMGDIPAAVNAFQEAASLLGKK

>sp|P49321|NASP_HUMAN/542-575

AQAHLKLGEVSVESENYVQAVEEFQSCLNLQEQY

>sp|P49321|NASP_HUMAN/584-617

AETHYQLGLAYGYNSQYDEAVAQFSKSIEVIENR

>sp|P49792|RBP2_HUMAN/60-93

PKAHRFLGLLYELEENTDKAVECYRRSVELNPTQ

>sp|P50502|F10A1_HUMAN/114-147

ANDKKVAAIEALNDGELQKAIDLFTDAIKLNPRL

>sp|P50502|F10A1_HUMAN/148-181

AILYAKRASVFVKLQKPNAAIRDCDRAIEINPDS

>sp|P50502|F10A1_HUMAN/182-215

AQPYKWRGKAHRLLGHWEEAAHDLALACKLDYDE

>sp|P50542|PEX5_HUMAN/488-521

PDVQCGLGVLFNLSGEYDKAVDCFTAALSVRPND

>sp|P50542|PEX5_HUMAN/522-555

YLLWNKLGATLANGNQSEEAVAAYRRALELQPGY

>sp|P50542|PEX5_HUMAN/369-402

MEAWQYLGTTQAENEQELLAISALRRCLELKPDN

>sp|P50542|PEX5_HUMAN/556-589

IRSRYNLGISCINLGAHREAVEHFLEALNMQRKS

>sp|P53041|PPP5_HUMAN/28-61

AEELKTQANDYFKAKDYENAIKFYSQAIELNPSN

>sp|P53041|PPP5_HUMAN/96-129

IKGYYRRAASNMALGKFRAALRDYETVVKVKPHD

>sp|P53041|PPP5_HUMAN/62-95

AIYYGNRSLAYLRTECYGYALGDATRAIELDKKY

>sp|P53804|TTC3_HUMAN/299-332

PKGHYRYCDALSMLGEYDWALQANIKAQKLCKND

>sp|P53804|TTC3_HUMAN/576-609

CLAYCGIGKVYLKKNRFLEALNHFEKARTLIYRL

>sp|P53804|TTC3_HUMAN/265-298

YLLYGNRALCFLRTGQFRNALGDGKRATILKNTW

>sp|P53804|TTC3_HUMAN/231-264

GELMKMKGNEEFSKERFDIAIIYYTRAIEYRPEN

>sp|P81274|GPSM2_HUMAN/322-355

GRACWSLGNAYTALGNHDQAMHFAEKHLEISREV

>sp|P81274|GPSM2_HUMAN/242-275

RRAYSNLGNAYIFLGEFETASEYYKKTLLLARQL

>sp|P81274|GPSM2_HUMAN/202-235

GRAFGNLGNTHYLLGNFRDAVIAHEQRLLIAKEF

>sp|P81274|GPSM2_HUMAN/282-315

AQSCYSLGNTYTLLQDYEKAIDYHLKHLAIAQEL

>sp|P81274|GPSM2_HUMAN/102-135

AKASGNLGNTLKVLGNFDEAIVCCQRHLDISREL

>sp|P81274|GPSM2_HUMAN/62-95

SAIYSQLGNAYFYLHDYAKALEYHHHDLTLARTI

>sp|Q02790|FKBP4_HUMAN/353-386

EKGLFRRGEAHLAVNDFELARADFQKVLQLYPNN

>sp|Q02790|FKBP4_HUMAN/319-352

LASHLNLAMCHLKLQAFSAAIESCNKALELDSNN

>sp|Q02790|FKBP4_HUMAN/270-303

STIVKERGTVYFKEGKYKQALLQYKKIVSWLEYE

>sp|Q07617|SPAG1_HUMAN/623-656

FKALKEEGNQCVNDKNYKDALSKYSECLKINNKE

>sp|Q07617|SPAG1_HUMAN/276-309

VKALLRRATTYKHQNKLREATEDLSKVLDVEPDN

>sp|Q07617|SPAG1_HUMAN/691-724

VKAFYRRALAHKGLKNYQKSLIDLNKVILLDPSI

>sp|Q07617|SPAG1_HUMAN/657-690

CAIYTNRALCYLKLCQFEEAKQDCDQALQLADGN

>sp|Q07617|SPAG1_HUMAN/209-242

ATREKEKGNEAFNSGDYEEAVMYYTRSISALPTV

>sp|Q07617|SPAG1_HUMAN/487-520

SILYSNRAACYLKEGNCSGCIQDCNRALELHPFS

>sp|Q07617|SPAG1_HUMAN/445-478

PAGLKSQGNELFRSGQFAEAAGKYSAAIALLEPA

>sp|Q07617|SPAG1_HUMAN/521-554

MKPLLRRAMAYETLEQYGKAYVDYKTVLQIDCGL

>sp|Q07866|KLC1_HUMAN/255-288

ATMLNILALVYRDQNKYKDAANLLNDALAIREKT

>sp|Q07866|KLC1_HUMAN/381-414

AKTKNNLASCYLKQGKFKQAETLYKEILTRAHER

>sp|Q07866|KLC1_HUMAN/297-330

AATLNNLAVLYGKRGKYKEAEPLCKRALEIREKV

>sp|Q07866|KLC1_HUMAN/464-497

TTTLKNLGALYRRQGKFEAAETLEEAAMRSRKQG

>sp|Q07866|KLC1_HUMAN/339-372

AKQLNNLALLCQNQGKYEEVEYYYQRALEIYQTK

>sp|Q08752|PPID_HUMAN/223-256

TEDLKNIGNTFFKSQNWEMAIKKYAEVLRYVDSS

>sp|Q08752|PPID_HUMAN/307-340

TKALYRRAQGWQGLKEYDQALADLKKAQGIAPED

>sp|Q08752|PPID_HUMAN/273-306

LSCVLNIGACKLKMSNWQGAIDSCLEALELDPSN

>sp|Q12797|ASPH_HUMAN/454-487

TSLKNDLGVGYLLIGDNDNAKKVYEEVLSVTPND

>sp|Q12797|ASPH_HUMAN/341-374

IKAELDAAEKLRKRGKIEEAVNAFKELVRKYPQS

>sp|Q13042|CDC16_HUMAN/368-401

HLPMLYIGLEYGLTNNSKLAERFFSQALSIAPED

>sp|Q13042|CDC16_HUMAN/402-435

PFVMHEVGVVAFQNGEWKTAEKWFLDALEKIKAI

>sp|Q13042|CDC16_HUMAN/130-163

SSICLLRGKIYDALDNRTLATYSYKEALKLDVYC

>sp|Q13042|CDC16_HUMAN/334-367

GPAWIAYGHSFAVESEHDQAMAAYFTAAQLMKGC

>sp|Q13042|CDC16_HUMAN/445-478

EPLLNNLGHVCRKLKKYAEALDYHRQALVLIPQN

>sp|Q13042|CDC16_HUMAN/479-512

ASTYSAIGYIHSLMGNFENAVDYFHTALGLRRDD

>sp|Q13099|IFT88_HUMAN/527-560

TEALYNIGLTYEKLNRLDEALDCFLKLHAILRNS

>sp|Q13099|IFT88_HUMAN/242-275

GILKMNMGNIYLKQRNYSKAIKFYRMALDQVPSV

>sp|Q13099|IFT88_HUMAN/561-594

AEVLYQIANIYELMENPSQAIEWLMQVVSVIPTD

>sp|Q13099|IFT88_HUMAN/595-628

PQVLSKLGELYDREGDKSQAFQYYYESYRYFPCN

>sp|Q13099|IFT88_HUMAN/281-314

IKIMQNIGVTFIQAGQYSDAINSYEHIMSMAPNL

>sp|Q13099|IFT88_HUMAN/663-696

VKWQLMVASCFRRSGNYQKALDTYKDTHRKFPEN

>sp|Q13099|IFT88_HUMAN/629-662

IEVIEWLGAYYIDTQFWEKAIQYFERASLIQPTQ

>sp|Q13099|IFT88_HUMAN/493-526

PAALTNKGNTVFANGDYEKAAEFYKEALRNDSSC

>sp|Q13099|IFT88_HUMAN/424-457

NDLEINKAVTYLRQKDYNQAVEILKVLEKKDSRV

>sp|Q13099|IFT88_HUMAN/459-492

SAAATNLSALYYMGKDFAQASSYADIAVNSDRYN

>sp|Q13217|DNJC3_HUMAN/71-104

YIAYYRRATVFLAMGKSKAALPDLTKVIQLKMDF

>sp|Q13217|DNJC3_HUMAN/37-70

VEKHLELGKKLLAAGQLADALSQFHAAVDGDPDN

>sp|Q13217|DNJC3_HUMAN/188-221

AELRELRAECFIKEGEPRKAISDLKAASKLKNDN

>sp|Q13217|DNJC3_HUMAN/340-373

VNALKDRAEAYLIEEMYDEAIQDYETAQEHNEND

>sp|Q13217|DNJC3_HUMAN/222-255

TEAFYKISTLYYQLGDHELSLSEVRECLKLDQDH

>sp|Q13217|DNJC3_HUMAN/105-138

TAARLQRGHLLLKQGKLDEAEDDFKKVLKSNPSE

>sp|Q13217|DNJC3_HUMAN/306-339

VRSKERICHCFSKDEKPVEAIRVCSEVLQMEPDN

>sp|Q13325|IFIT5_HUMAN/249-282

PYVLRYAAKFYRRKNSWNKALELLKKALEVTPTS

>sp|Q13325|IFIT5_HUMAN/51-84

LALYNLLAYVKHLKGQNKDALECLEQAEEIIQQE

>sp|Q13325|IFIT5_HUMAN/435-468

VQSLSALGFVYKLEGEKRQAAEYYEKAQKIDPEN

>sp|Q13325|IFIT5_HUMAN/338-371

AFAYTDLANMYAEGGQYSNAEDIFRKALRLENIT

>sp|Q13325|IFIT5_HUMAN/140-173

TDCEKGWALLKFGGKYYQKAKAAFEKALEVEPDN

>sp|Q13451|FKBP5_HUMAN/317-350

LAAFLNLAMCYLKLREYTKAVECCDKALGLDSAN

>sp|Q13451|FKBP5_HUMAN/351-384

EKGLYRRGEAQLLMNEFESAKGDFEKVLEVNPQN

>sp|Q13702|RAPSN_HUMAN/206-239

AMSQYHMAVAYRLLGRLGSAMECCEESMKIALQH

>sp|Q13702|RAPSN_HUMAN/286-319

VQALLGVAKCWVARKALDKALDAIERAQDLAEEV

>sp|Q13702|RAPSN_HUMAN/6-39

TKQQIEKGLQLYQSNQTEKALQVWTKVLEKSSDL

>sp|Q13702|RAPSN_HUMAN/83-116

LESYLNLARSNEKLCEFHKTISYCKTCLGLPGTR

>sp|Q13702|RAPSN_HUMAN/123-156

GQVSLSMGNAFLGLSVFQKALESFEKALRYAHNN

>sp|Q13702|RAPSN_HUMAN/246-279

ALCLLCFADIHRSRGDLETAFPRYDSAMSIMTEI

>sp|Q13702|RAPSN_HUMAN/163-196

CRVCCSLGSFYAQVKDYEKALFFPCKAAELVNNY

>sp|Q14318|FKBP8_HUMAN/228-261

GNAHYQRADFVLAANSYDLAIKAITSSAKVDMTF

>sp|Q14318|FKBP8_HUMAN/272-305

VKCLNNLAASQLKLDHYRAALRSCSLVLEHQPDN

>sp|Q14318|FKBP8_HUMAN/306-339

IKALFRKGKVLAQQGEYSEAIPILRAALKLEPSN

>sp|Q15006|EMC2_HUMAN/87-120

HRVKRLTGMRFEAMERYDDAIQLYDRILQEDPTN

>sp|Q15006|EMC2_HUMAN/192-225

CQQYAEVKYTQGGLENLELSRKYFAQALKLNNRN

>sp|Q15006|EMC2_HUMAN/155-188

QEAWHELAELYINEHDYAKAAFCLEELMMTNPHN

>sp|Q15785|TOM34_HUMAN/193-226

ARVLKEEGNELVKKGNHKKAIEKYSESLLCSNLE

>sp|Q15785|TOM34_HUMAN/261-294

VKAFYRRAQAHKALKDYKSSFADISNLLQIEPRN

>sp|Q15785|TOM34_HUMAN/85-118

IKPLLRRASAYEALEKYPMAYVDYKTVLQIDDNV

>sp|Q15785|TOM34_HUMAN/227-260

SATYSNRALCYLVLKQYTEAVKDCTEALKLDGKN

>sp|Q15785|TOM34_HUMAN/51-84

SVLYSNRAACHLKDGNCRDCIKDCTSALALVPFS

>sp|Q15785|TOM34_HUMAN/9-42

VEELRAAGNESFRNGQYAEASALYGRALRVLQAQ

>sp|Q17RB8|LONF1_HUMAN/48-81

WELLLRRGELLALGGHLKGALEAFAAALRRGAPA

>sp|Q17RB8|LONF1_HUMAN/279-312

PEVYFRKGKVLCDAGFLGDALQLFLQCLALDEDF

>sp|Q17RB8|LONF1_HUMAN/245-278

LIVKIYRAESYAGLQEFKAAIEDLNAVLFQLPDW

>sp|Q1L5Z9|LONF2_HUMAN/231-264

NSLLLLRAELYLTMKNYEQALQDASAACQNEPLL

>sp|Q1L5Z9|LONF2_HUMAN/197-230

LRRLAGQARSLQRQQQPEAALLRCDQALELAPDD

>sp|Q1L5Z9|LONF2_HUMAN/265-298

IKGHQVKAQALSGLGRSKEVLKEFLYCLALNPEC

>sp|Q496Y0|LONF3_HUMAN/243-276

ASQLRHEGNRLYRERQVEAALLKYNEAVKLAPND

>sp|Q496Y0|LONF3_HUMAN/311-344

FKAHFRKAQALATLGKVEEALREFLYCVSLDGKN

>sp|Q496Y0|LONF3_HUMAN/67-100

SKVLLTQADALASRGRIREALEVYRQLSERQQLV

>sp|Q496Y0|LONF3_HUMAN/277-310

HLLYSNRSQIYFTLESHENALHDAEIACKLRPMG

>sp|Q49AM3|TTC31_HUMAN/373-406

PRGLFRLGKALMGLQRFREAAAVFQETLRGGSQP

>sp|Q49AM3|TTC31_HUMAN/305-338

SQELAKLGTSFAQNGFYHEAVVLFTQALKLNPQD

>sp|Q49AM3|TTC31_HUMAN/339-372

HRLFGNRSFCHERLGQPAWALADAQVALTLRPGW

>sp|Q5I0X7|TTC32_HUMAN/58-91

ATAYNNRGQIKYFRVDFYEAMDDYTSAIEVQPNF

>sp|Q5I0X7|TTC32_HUMAN/92-125

EVPYYNRGLILYRLGYFDDALEDFKKVLDLNPGF

>sp|Q5SRH9|TT39A_HUMAN/315-348

AIFLFFAGRIEVIKGNIDAAIRRFEECCEAQQHW

>sp|Q5SRH9|TT39A_HUMAN/546-579

PNALLELALLLMEQDRNEEAIKLLESAKQNYKNY

>sp|Q5SRH9|TT39A_HUMAN/505-538

CLVKLLKGLCLKYLGRVQEAEENFRSISANEKKI

>sp|Q5T0N1|CFA70_HUMAN/1000-1033

HFIFLRLGLIYLEEKEYEKAKKTYMQACKRSPSC

>sp|Q5T0N1|CFA70_HUMAN/669-702

LDHWLDYGAFCLLTEDNIKAQECFQKALSLNQSH

>sp|Q5T0N1|CFA70_HUMAN/737-770

VVAWTLLGLYYEIQNNDIRMEMAFHEASKQLQAR

>sp|Q5T0N1|CFA70_HUMAN/963-996

PNVWGLKGHLYFLSGNHSEAKACYERTISFVVDA

>sp|Q5T0N1|CFA70_HUMAN/635-668

SEQLQLFAFEAEVNENFEMAAAYYKERLVREPQN

>sp|Q5T0N1|CFA70_HUMAN/703-736

IHSLLLCGVLAVLLENYEQAEIFFEDATCLEPTN

>sp|Q5T0N1|CFA70_HUMAN/929-962

CEYYLVLAQTHILKKNFAKAEEYLQQAAQMDYLN

>sp|Q5T0N1|CFA70_HUMAN/1067-1100

AEVWAYLALVCLKVGRQLEAEQAYKYMIKLKLKD

>sp|Q5T4D3|TMTC4_HUMAN/550-583

AAAWMNLGIVQNSLKRFEAAEQSYRTAIKHRRKY

>sp|Q5T4D3|TMTC4_HUMAN/584-617

PDCYYNLGRLYADLNRHVDALNAWRNATVLKPEH

>sp|Q5T4D3|TMTC4_HUMAN/686-719

ASYHGNLAVLYHRWGHLDLAKKHYEISLQLDPTA

>sp|Q5T4D3|TMTC4_HUMAN/652-685

HSLMFSLANVLGKSQKYKESEALFLKAIKANPNA

>sp|Q5T4D3|TMTC4_HUMAN/482-515

AKVHYNIGKNLADKGNQTAAIRYYREAVRLNPKY

>sp|Q5T4D3|TMTC4_HUMAN/516-549

VHAMNNLGNILKERNELQEAEELLSLAVQIQPDF

>sp|Q5T4D3|TMTC4_HUMAN/618-651

SLAWNNMIILLDNTGNLAQAEAVGREALELIPND

>sp|Q5T764|IFT1B_HUMAN/437-470

VESVSLLGLIHKLKGEVSDALLCYERALRLAADL

>sp|Q5T764|IFT1B_HUMAN/141-174

VDCEEGWALAKCGGKNYERAKTCFEKALEGNPEN

>sp|Q5T764|IFT1B_HUMAN/251-284

AYVFQYAAKFYRRKGSVDKALELLKMALETTPTS

>sp|Q5T764|IFT1B_HUMAN/52-85

VGIHNLLAYVKHLKGQNEEALVSLKKAEDLIQKE

>sp|Q5T764|IFT1B_HUMAN/340-373

EMAYVDLAETYAEIGHHRKAEEHFQKGLRMKIFE

>sp|Q5T764|IFT1B_HUMAN/95-128

LVTWGNFAWVYYHMGRLAEAQTYLDKVENTCKKF

>sp|Q5TAA0|TTC22_HUMAN/295-328

PPILNRLAKIFYFLGKQDMAIGTCNMALDVLRDP

>sp|Q5TAA0|TTC22_HUMAN/432-465

PELQLLRGKCLRIKGEDANAAACFKRAVELDDAG

>sp|Q5TAA0|TTC22_HUMAN/66-99

PAVRHLLGAFAFYLEELDEARECFLEVAHEHPGN

>sp|Q5VTQ0|TT39B_HUMAN/585-618

CLVKLLKGCCLKNLQRPLQAELCYNHVVESEKLL

>sp|Q5VTQ0|TT39B_HUMAN/393-426

SLVLFYHARIELLKGNLEEAQEVFQKCISVQEEW

>sp|Q5VTQ0|TT39B_HUMAN/626-659

PFTLFELASLYKSQGEIDKAIKFLETARNNYKDY

>sp|Q5W5X9|TTC23_HUMAN/186-219

ARIRLSFAQVYQGQKKSKEALSHYQAALEYVEIS

>sp|Q5W5X9|TTC23_HUMAN/356-389

AETYRLLGGADLAQGNHSGARKKLKKCLQIQTLL

>sp|Q5W5X9|TTC23_HUMAN/137-170

IELFHTMGRALLSLQKFKEAAENLTKAERLSKEL

>sp|Q6DKK2|TTC19_HUMAN/237-270

GMCLDACARYLLFSKQPSQAQRMYEKALQISEEI

>sp|Q6DKK2|TTC19_HUMAN/318-351

HMVLSNLAAVLMHRERYTQAKEIYQEALKQAKLK

>sp|Q6DKK2|TTC19_HUMAN/96-129

IIQLLKRAKLSIMKDEPEEAELILHDALRLAYQT

>sp|Q6DKK2|TTC19_HUMAN/279-312

IVLMSDLATTLDAQGRFDEAYIYMQRASDLARQI

>sp|Q6N069|NAA16_HUMAN/80-113

HVCWHVYGLLQRSDKKYDEAIKCYRNALKLDKDN

>sp|Q6N069|NAA16_HUMAN/408-441

IELFYMKAKIYKHIGNLKEAAKWMDEAQSLDTAD

>sp|Q6N069|NAA16_HUMAN/672-705

IDTHLLAFEIYFRKGKFLLMLQSVKRAFAINSNN

>sp|Q6N069|NAA16_HUMAN/224-257

LLVEEIKGEILLKLGRLKEASEVFKNLIDRNAEN

>sp|Q6N069|NAA16_HUMAN/374-407

LWVQYFLAQHFDKLGQYSLALDYINAAIASTPTL

>sp|Q6N069|NAA16_HUMAN/46-79

GETLAMKGLTLNCLGKKEEAYEFVRKGLRNDVKS

>sp|Q6P3X3|TTC27_HUMAN/561-594

LGVWFSLGCAYLALEDYQGSAKAFQRCVTLEPDN

>sp|Q6P3X3|TTC27_HUMAN/595-628

AEAWNNLSTSYIRLKQKVKAFRTLQEALKCNYEH

>sp|Q6P3X3|TTC27_HUMAN/629-662

WQIWENYILTSTDVGEFSEAIKAYHRLLDLRDKY

>sp|Q6P3X3|TTC27_HUMAN/527-560

ARAQRSKALLHLRNKEFQECVECFERSVKINPMQ

>sp|Q6P597|KLC3_HUMAN/333-366

AKQLNNLALLCQNQGKFEDVERHYARALSIYEAL

>sp|Q6P597|KLC3_HUMAN/375-408

AKTKNNLASAYLKQNKYQQAEELYKEILHKEDLP

>sp|Q6P597|KLC3_HUMAN/291-324

AATLNNLAVLYGKRGRYREAEPLCQRALEIREKV

>sp|Q6P597|KLC3_HUMAN/249-282

ATMLNILALVYRDQNKYKEATDLLHDALQIREQT

>sp|Q6PD62|CTR9_HUMAN/531-564

VDCYLRLGAMARDKGNFYEASDWFKEALQINQDH

>sp|Q6PD62|CTR9_HUMAN/681-714

SDVWLNLAHIYVEQKQYISAVQMYENCLRKFYKH

>sp|Q6PD62|CTR9_HUMAN/451-484

PEILNNVGALHFRLGNLGEAKKYFLASLDRAKAE

>sp|Q6PD62|CTR9_HUMAN/497-530

VTTSYNLARLYEAMCEFHEAEKLYKNILREHPNY

>sp|Q6PD62|CTR9_HUMAN/565-598

PDAWSLIGNLHLAKQEWGPGQKKFERILKQPSTQ

>sp|Q6PD62|CTR9_HUMAN/717-750

TEVVLYLARALFKCGKLQECKQTLLKARHVAPSD

>sp|Q6PD62|CTR9_HUMAN/341-374

VLPFFGLGQMYIYRGDKENASQCFEKVLKAYPNN

>sp|Q6PD62|CTR9_HUMAN/198-231

AEVRLGMGHCFVKLNKLEKARLAFSRALELNSKC

>sp|Q6PD62|CTR9_HUMAN/163-196

IPALLGKACISFNKKDYRGALAYYKKALRTNPGC

>sp|Q6PD62|CTR9_HUMAN/306-339

AESCYQLARSFHVQEDYDQAFQYYYQATQFASSS

>sp|Q6PGP7|TTC37_HUMAN/598-631

FNCWESLGEAYLSRGGYTTALKSFTKASELNPES

>sp|Q6PGP7|TTC37_HUMAN/306-339

TSGWYHLAEAQVKMHRPKEAVLSCSQALKIVDNL

>sp|Q6PGP7|TTC37_HUMAN/6-39

VKTALKSARDAIRNKEYKEALKHCKTVLKQEKNN

>sp|Q6PGP7|TTC37_HUMAN/272-305

GPGLIGLGIKALQDKKYEDAVRNLTEGLKESPVC

>sp|Q6PGP7|TTC37_HUMAN/980-1013

APAFTMLGYLNEHLQLKKEAANAYQRAILLLQTA

>sp|Q6PGP7|TTC37_HUMAN/1051-1084

LEDIIGFALALFMKGLYKESSKAYERALSIVESE

>sp|Q6PGP7|TTC37_HUMAN/1400-1433

VPAWQWLAHVYQSQGMMRAAEMCYRKSLQLASQR

>sp|Q6PGP7|TTC37_HUMAN/40-73

YNAWVFIGVAAAELEQPDQAQSAYKKAAELEPDQ

>sp|Q6PGP7|TTC37_HUMAN/861-894

AVAWTNLGVLYLTNENIEQAHEAFKMAQSLDPSY

>sp|Q6PGP7|TTC37_HUMAN/564-597

KWAWLRRGLYYLKAGQHSQAVADLQAALRADPKD

>sp|Q6PGP7|TTC37_HUMAN/420-453

AEVHALEALIHFTKKDYLQAEKCFQRALEKDTEV

>sp|Q6PGP7|TTC37_HUMAN/632-665

IYSVFKVAAIQQILGKYKEAVAQYQMIIKKKEDY

>sp|Q6PID6|TTC33_HUMAN/127-160

WESWQTLGRAQLGLGEIILAIRSFQVALHIYPMN

>sp|Q6PID6|TTC33_HUMAN/59-92

SKQLKDEGASLAENKRYREAIQKWDEALQLTPND

>sp|Q6ZXV5|TMTC3_HUMAN/703-736

RSALFNLALLYSQTAKELKALPILEELLRYYPDH

>sp|Q6ZXV5|TMTC3_HUMAN/480-513

IGAHMNVGRTYKNLNRTKEAEESYMMAKSLMPQI

>sp|Q6ZXV5|TMTC3_HUMAN/446-479

AKLWNNVGHALENEKNFERALKYFLQATHVQPDD

>sp|Q6ZXV5|TMTC3_HUMAN/669-702

ANGYFNLGMLAMDDKKDNEAEIWMKKAIKLQADF

>sp|Q6ZXV5|TMTC3_HUMAN/772-805

VQGKHNLCVVYFEEKDLLKAERCLLETLALAPHE

>sp|Q6ZXV5|TMTC3_HUMAN/563-596

KQAYISRGELLLKMNKPLKAKEAYLKALELDRNN

>sp|Q7Z3J3|RGPD4_HUMAN/60-93

PRAHRFLGLLYELEENTEKAVECYRRSVELNPTQ

>sp|Q7Z494|NPHP3_HUMAN/943-976

ADLYETLGRFLKDLGLLSQAIVPLQRSLEIRETA

>sp|Q7Z494|NPHP3_HUMAN/985-1018

AQSLHQLASVYVQWKKFGNAEQLYKQALEISENA

>sp|Q7Z494|NPHP3_HUMAN/1027-1060

ARELEALATLYQKQNKYEQAEHFRKKSFKIHQKA

>sp|Q7Z494|NPHP3_HUMAN/1093-1126

ARTLNELGVLYYLQNNLETADQFLKRSLEMRERV

>sp|Q7Z494|NPHP3_HUMAN/1177-1210

AYTVKHLAILYKKMGKLDKAVPLYELAVEIRQKS

>sp|Q7Z494|NPHP3_HUMAN/1261-1294

GETLKNLAVLSYEGGDFEKAAELYKRAMEIKEAE

>sp|Q7Z494|NPHP3_HUMAN/1135-1168

AQSLNNLAALCNEKKQYDKAEELYERALDIRRRA

>sp|Q7Z494|NPHP3_HUMAN/1219-1252

ATALVNLAVLYSQMKKHVEALPLYERALKIYEDS

>sp|Q7Z4L5|TT21B_HUMAN/563-596

PLYHLIKAQSQKKMGEIADAIKTLHMAMSLPGMK

>sp|Q7Z4L5|TT21B_HUMAN/756-789

GTLASKMGKALIKTHNYSMAITYYEAALKTGQKN

>sp|Q7Z4L5|TT21B_HUMAN/108-141

EKALYHAGLFLWHIGRHDKAREYIDRMIKISDGS

>sp|Q7Z4L5|TT21B_HUMAN/884-917

AEICAEIAKHSVAQRDYEKAIKFYREALVHCETD

>sp|Q7Z4L5|TT21B_HUMAN/722-755

PRSFLLLGDAYMNILEPEEAIVAYEQALNQNPKD

>sp|Q7Z4L5|TT21B_HUMAN/492-525

LQTVFLIAKVKYLSGDIEAAFNNLQHCLEHNPSY

>sp|Q7Z4L5|TT21B_HUMAN/654-687

VRVTIANADLALAQGDIERALSILQNVTAEQPYF

>sp|Q7Z4L5|TT21B_HUMAN/1157-1190

IPALLGMATAYMILKQTPRARNQLKRIAKMNWNA

>sp|Q7Z4L5|TT21B_HUMAN/178-211

TFALLGKAQCLEMRQNYSGALETVNQIIVNFPSF

>sp|Q7Z4L5|TT21B_HUMAN/526-559

ADAHLLLAQVYLSQEKVKLCSQSLELCLSYDFKV

>sp|Q7Z4L5|TT21B_HUMAN/952-985

EAATMMMADLMFRKQDYEQAVFHLQQLLERKPDN

>sp|Q7Z4L5|TT21B_HUMAN/324-357

SEFATELGYQMILQGRVKEALKWYKTAMTLDETS

>sp|Q7Z4L5|TT21B_HUMAN/1197-1230

EKSWLLLADIYIQSAKYDMAEDLLKRCLRHNRSC

>sp|Q7Z4L5|TT21B_HUMAN/918-951

NKIMLELARLYLAQDDPDSCLRQCALLLQSDQDN

>sp|Q7Z4L5|TT21B_HUMAN/831-864

GRCQVLLAKVYSKMEKLGDAITALQQARELQARV

>sp|Q7Z4L5|TT21B_HUMAN/1266-1299

PAVGYKLAFNYLKAKRYVDSIDICHQVLEAHPTY

>sp|Q86TV6|TTC7B_HUMAN/515-548

HQAAFYLALQLAISRQIPEALGYVRQALQLQGDD

>sp|Q86TV6|TTC7B_HUMAN/798-831

HEVWNGLGEVLQAQGNDAAATECFLTALELEASS

>sp|Q86TV6|TTC7B_HUMAN/764-797

VKSMQRLALILHQLGRYSLAEKILRDAVQVNSTA

>sp|Q86TV6|TTC7B_HUMAN/696-729

AQIWLHAAEVYIGIGKPAEATACTQEAANLFPMS

>sp|Q86TV6|TTC7B_HUMAN/730-763

HNVLYMRGQIAELRGSMDEARRWYEEALAISPTH

>sp|Q86TV6|TTC7B_HUMAN/549-582

ANSLHLLALLLSAQKHYHDALNIIDMALSEYPEN

>sp|Q86TV6|TTC7B_HUMAN/397-430

FHLWYQFALSLMAAGKSARAVKVLKECIRLKPDD

>sp|Q86TZ1|TTC6_HUMAN/484-517

ALVYNFRAKVRGKIGLIEEAMADYNQALDLEDYA

>sp|Q86TZ1|TTC6_HUMAN/210-243

GSLCHATAMCHHRINEFEEAVNFFTWALKINPCF

>sp|Q86TZ1|TTC6_HUMAN/281-314

IKARISFGYNLQAQGKFQKAWNHFTIAIDTDPKN

>sp|Q86TZ1|TTC6_HUMAN/348-381

AEFLTNRGVIHEFMGHKQNAMKDYQDAITLNPKY

>sp|Q86TZ1|TTC6_HUMAN/382-415

SLAYFNAGNIYFHHRQFSQASDYFSKALKFDPEN

>sp|Q86TZ1|TTC6_HUMAN/450-483

AAVYFNRAHFYYCLKQYELAEEDLNKALSLKPND

>sp|Q86TZ1|TTC6_HUMAN/416-449

EYVLMNRAITNTILKKYEEAKEDFANVIESCPFW

>sp|Q86TZ1|TTC6_HUMAN/139-172

YTAFYNRALCYTKIRELQMALTDYGIVLLLDATE

>sp|Q86TZ1|TTC6_HUMAN/176-209

LNTFLNRGLIYVELGQYGFALEDFKQAALISRTN

>sp|Q86TZ1|TTC6_HUMAN/57-90

MTMCALLAKVQMKAKRTKEAVEVLKKALDAISHS

>sp|Q86UR1|NOXA1_HUMAN/122-155

WEVLHNVASAQCQLGLWTEAASSLREAMSKWPEG

>sp|Q86UR1|NOXA1_HUMAN/38-71

ARLCFNAGCVHLLAGDPEAALRAFDQAVTKDTCM

>sp|Q86UR1|NOXA1_HUMAN/72-105

AVGFFQRGVANFQLARFQEALSDFWLALEQLRGH

>sp|Q86WT1|TT30A_HUMAN/154-187

TDGQVNLGCLLYKEGQYEAACSKFSATLQASGYQ

>sp|Q86WT1|TT30A_HUMAN/45-78

RAGLSLLGYCYYRLQEFALAAECYEQLGQLHPEL

>sp|Q86WT1|TT30A_HUMAN/424-457

IPVLMAQAKIYWNLENYPMVEKVFRKSVEFCNDH

>sp|Q86WT1|TT30A_HUMAN/188-221

PDLSYNLALAYYSSRQYASALKHIAEIIERGIRQ

>sp|Q86YR5|GPSM1_HUMAN/66-99

SAIYSQLGNAYFYLKEHGRALEYHKHDLLLARTI

>sp|Q86YR5|GPSM1_HUMAN/209-242

GRAYGNLGNTHYLLGNFTEATTFHKERLAIAKEF

>sp|Q86YR5|GPSM1_HUMAN/289-322

AQACYSLGNTYTLLQDYERAAEYHLRHLLIAQEL

>sp|Q86YR5|GPSM1_HUMAN/106-139

AKASGNLGNTLKVLGRFDEAAVCCQRHLSIAQEQ

>sp|Q86YR5|GPSM1_HUMAN/329-362

GRACWSLGNAYVSMGRPAQALTFAKKHLQISQEI

>sp|Q86YR5|GPSM1_HUMAN/249-282

RRAYSNLGNAHVFLGRFDVAAEYYKKTLQLSRQL

>sp|Q8IUR5|TMTC1_HUMAN/548-581

NRALFNLGNLLKSQEKKEEAITLLKDSIKYGPEF

>sp|Q8IUR5|TMTC1_HUMAN/789-822

SELFFTKGNQLREQNLLDKAFESYRVAVQLNPDQ

>sp|Q8IUR5|TMTC1_HUMAN/483-516

AKVHYNYANFLKDQGRNKEAIYHYRTALKLYPRH

>sp|Q8IUR5|TMTC1_HUMAN/751-784

LECYRLLSAIYSKQENHDKALDAIDKALQLKPKD

>sp|Q8IUR5|TMTC1_HUMAN/616-649

SDLHNNYGVFLVDTGLPEKAVAHYQQAIKLSPSH

>sp|Q8IUR5|TMTC1_HUMAN/582-615

ADAYSSLASLLAEQERFKEAEEIYQTGIKNCPDS

>sp|Q8IUR5|TMTC1_HUMAN/650-683

HVAMVNLGRLYRSLGENSMAEEWYKRALQVAHKA

>sp|Q8IUR5|TMTC1_HUMAN/823-856

AQAWMNMGGIQHIKGKYVSARAYYERALQLVPDS

>sp|Q8IWX7|UN45B_HUMAN/6-39

AVQLKEEGNRHFQLQDYKAATNSYSQALKLTKDK

>sp|Q8IWX7|UN45B_HUMAN/43-76

ATLYRNRAACGLKTESYVQAASDASRAIDINSSD

>sp|Q8IWX7|UN45B_HUMAN/77-110

IKALYRRCQALEHLGKLDQAFKDVQRCATLEPRN

>sp|Q8IYB4|PEX5R_HUMAN/542-575

IRSRYNLGISCINLGAYREAVSNFLTALSLQRKS

>sp|Q8IYB4|PEX5R_HUMAN/394-427

LKALMALAVSYTNTGHQQDACDALKNWIKQNPKY

>sp|Q8IYB4|PEX5R_HUMAN/360-393

AEAWQFLGITQAENENEQAAIVALQRCLELQPNN

>sp|Q8IYB4|PEX5R_HUMAN/508-541

YSLWNRLGATLANGDRSEEAVEAYTRALEIQPGF

>sp|Q8IYB4|PEX5R_HUMAN/474-507

PDLQTGLGVLFHLSGEFNRAIDAFNAALTVRPED

>sp|Q8IYF3|TEX11_HUMAN/457-490

TKLQRNMACCYLNLQQLDKAKEAVAEAERHDPRN

>sp|Q8IYF3|TEX11_HUMAN/417-450

HNILWRQAASSFEVQNYTDALQWYYYSLRFYSTD

>sp|Q8IZP2|ST134_HUMAN/144-177

AILYAKRASVFVKLQKPNAAIRDCDRAIEINPDS

>sp|Q8IZP2|ST134_HUMAN/178-211

AQPYKRRGKAHRLLGHWEEAAHDLALACKFDYDE

>sp|Q8IZP2|ST134_HUMAN/110-143

ANDKKVAAIEALNDGELQKAIDLFTDAIKLNPRL

>sp|Q8N0Z6|TTC5_HUMAN/224-257

PDLHLNRATLHKYEESYGEALEGFSRAAALDPAW

>sp|Q8N0Z6|TTC5_HUMAN/103-136

VEAWNQLGEVYWKKGDVAAAHTCFSGALTHCRNK

>sp|Q8N394|TMTC2_HUMAN/493-526

AKAWGNLGNVLKSQSKISEAESAYRNALYYRSNM

>sp|Q8N394|TMTC2_HUMAN/677-710

IPAHLTYGKLLALTGRKSEAEKLFLKAIELDPTK

>sp|Q8N394|TMTC2_HUMAN/561-594

ASAYLNTGIILMNQGRTEEARRTFLKCSEIPDEN

>sp|Q8N394|TMTC2_HUMAN/643-676

QSLYNMMGEAYMRLSKLPEAEHWYMESLRSKTDH

>sp|Q8N394|TMTC2_HUMAN/606-639

TSCLYNLGKLYHEQGHYEEALSVYKEAIQKMPRQ

>sp|Q8N394|TMTC2_HUMAN/527-560

ADMLYNLGLLLQENSRFAEALHYYKLAIGSRPTL

>sp|Q8N394|TMTC2_HUMAN/745-778

FDVVFNAAHMLRQASLNEAAEKYYDLAARLRPNY

>sp|Q8N394|TMTC2_HUMAN/779-812

PAALMNLGAILHLNGRLQKAEANYLRALQLKPDD

>sp|Q8N4P2|TT30B_HUMAN/424-457

IPVLMAQAKIYWNLENYPMVEKIFRKSVEFCNDH

>sp|Q8N4P2|TT30B_HUMAN/45-78

RAGLSLLGYCYYRLQEFALAAECYEQLGQLHPEL

>sp|Q8N4P2|TT30B_HUMAN/154-187

TDGQINLGCLLYKEGQYEAACSKFFAALQASGYQ

>sp|Q8N4P2|TT30B_HUMAN/188-221

PDLSYNLALAYYSSRQYASALKHIAEIIERGIRQ

>sp|Q8N5D0|WDTC1_HUMAN/362-395

LERVKQQANEAFACQQWTQAIQLYSKAVQRAPHN

>sp|Q8N5D0|WDTC1_HUMAN/433-466

LKAHFRLARCLFELKYVAEALECLDDFKGKFPEQ

>sp|Q8N5M4|TTC9C_HUMAN/8-41

AQLYKEEGNQRYREGKYRDAVSRYHRALLQLRGL

>sp|Q8N5M4|TTC9C_HUMAN/108-141

AKALYRAGVAFFHLQDYDQARHYLLAAVNRQPKD

>sp|Q8N6N2|TTC9B_HUMAN/171-204

FKATYRAGIAFYHLGDYARALRYLQEARSREPTD

>sp|Q8N6N2|TTC9B_HUMAN/65-98

AVAFKAEGQRCYREKKFREAIGKYHRALLQLKAA

>sp|Q8NA56|TTC29_HUMAN/274-307

AEASYYLGLAHLAAEEYETALTVLDTYCKISTDL

>sp|Q8NA56|TTC29_HUMAN/354-387

VRASTMLGDIYNEKGYYNKASECFQQAFDTTVEL

>sp|Q8NA56|TTC29_HUMAN/182-215

AEAHMHMGLLYEEDGQLLEAAEHYEAFHQLTQGR

>sp|Q8NA56|TTC29_HUMAN/314-347

GRGYEAIAKVLQSQGEMTEAIKYLKKFVKIARNN

>sp|Q8NBP0|TTC13_HUMAN/351-384

VQTLQLRGMMLYHHGSLQEALKNFKRCLQLEPYN

>sp|Q8NBP0|TTC13_HUMAN/283-316

PIAMLYKGLTFFHRGLLKEAIESFKEALKQKVDF

>sp|Q8NBP0|TTC13_HUMAN/143-176

TNEELAIAYVLIGSGLYDEAIRHFSTMLQEEPDL

>sp|Q8NBP0|TTC13_HUMAN/317-350

IDAYKSLGQAYRELGNFEAATESFQKALLLNQNH

>sp|Q8NBP0|TTC13_HUMAN/216-249

PEVFEQRAEILSPLGRINEAVNDLTKAIQLQPSA

>sp|Q8NDW8|TT21A_HUMAN/1270-1303

PAIGFKLAFNYLKDKKFVEAIEICNDVLREHPDY

>sp|Q8NDW8|TT21A_HUMAN/110-143

GTALYYAGLFLWLIGRHDKAKEYIDRMLKISRGF

>sp|Q8NDW8|TT21A_HUMAN/889-922

ASICIQFAEHYLAEKEYDKAVQSYKDVFSYLPTD

>sp|Q8NDW8|TT21A_HUMAN/572-605

PLYHLIKARALNKAGDYPEAIKTLKMVIKLPALK

>sp|Q8NDW8|TT21A_HUMAN/957-990

ETASVLMADLMFRKQKHEAAINLYHQVLEKAPDN

>sp|Q8NDW8|TT21A_HUMAN/923-956

NKVMLELAQLYLLQGHLDLCEQHCAILLQTEQNH

>sp|Q8NDW8|TT21A_HUMAN/762-795

ASLASRIGHAYVKAHQYTEAIEYYEAAQKINGQD

>sp|Q8NDW8|TT21A_HUMAN/1201-1234

EKSWLLLADIYCQGSKFDLALELLRRCVQYNKSC

>sp|Q8NDW8|TT21A_HUMAN/728-761

PHTSLLLGDALMSILEPEKALEVYDEAYRQNPHD

>sp|Q8NDW8|TT21A_HUMAN/180-213

VLGLMGKAMYFMMQQNYSEALEVVNQITVTSGSF

>sp|Q8NDW8|TT21A_HUMAN/334-367

VHVATELGYLFILKNQVKEALLWYSEAMKLDKDG

>sp|Q8NEE8|TTC16_HUMAN/95-128

VDFYALRAEAYLQLCDFSSAAQNLRRAYSLQQDN

>sp|Q8NEE8|TTC16_HUMAN/136-169

TFVLYLQGQCLFEQCAFLDALNVFSHAAELQPEK

>sp|Q8NEE8|TTC16_HUMAN/251-284

AQQARQDAGILAVQGKLQHALQRINRAIENNPLD

>sp|Q8NEE8|TTC16_HUMAN/406-439

GLLQEKMGFCEQRRKQFQKAENHFSTAIRHNPQK

>sp|Q8NEE8|TTC16_HUMAN/365-398

KGLYINRGDCFFQLGNLAFAEADYQQALALSPQD

>sp|Q8NEE8|TTC16_HUMAN/285-318

PSLFLFRGTMYRRLQEFDGAVEDFLKVLDMVTED

>sp|Q8NEE8|TTC16_HUMAN/61-94

VREYYSRGQQCLEQADWETAVLLFSRALHLDPQL

>sp|Q8NEE8|TTC16_HUMAN/440-473

AQYYLYRAKSRQLLQNIFGARQDVATVLLLNPKQ

>sp|Q8NEE8|TTC16_HUMAN/204-237

ADVYIFRARLYNFLQKPHLCYRDLHSALLLNPKH

>sp|Q8NFI4|F10A5_HUMAN/114-147

ANDKKVAAIEVLNDGELQKAIDLFTDAIKLNPRL

>sp|Q8NFI4|F10A5_HUMAN/148-181

AILYAKRASVFVKLQKPNAAIQDCDRAIEINPDS

>sp|Q8NFI4|F10A5_HUMAN/182-215

AQPYKWRGKAHRLLGHWEEAAHDLAFACKLDYDE

>sp|Q8TAM2|TTC8_HUMAN/457-490

AEAYNNLAVLEMRKGHVEQARALLQTASSLAPHM

>sp|Q8TAM2|TTC8_HUMAN/352-385

VEAIACIGSNHFYSDQPEIALRFYRRLLQMGIYN

>sp|Q8TAM2|TTC8_HUMAN/491-524

YEPHFNFATISDKIGDLQRSYVAAQKSEAAFPDH

>sp|Q8TAM2|TTC8_HUMAN/423-456

ADVWYNLGHVAVGIGDTNLAHQCFRLALVNNNNH

>sp|Q8TAM2|TTC8_HUMAN/251-284

WWWKVQIGKCYYRLGMYREAEKQFKSALKQQEMV

>sp|Q8TAM2|TTC8_HUMAN/318-351

VTLLCGIARIYEEMNNMSSAAEYYKEVLKQDNTH

>sp|Q8TAM2|TTC8_HUMAN/386-419

GQLFNNLGLCCFYAQQYDMTLTSFERALSLAENE

>sp|Q8TE82|S3TC1_HUMAN/601-634

VAVYANLASIYRKQKNREKCAQVVPKAMALLLGT

>sp|Q8TE82|S3TC1_HUMAN/560-593

ARLCFLLGRLCSRRLKLSQARVYFEEALGALEGS

>sp|Q8TE82|S3TC1_HUMAN/863-896

GVIANMVAVALKRTGRTRQAAESYYRALRVARDL

>sp|Q8TE82|S3TC1_HUMAN/665-698

ARACFLLARHHVHLKQPEEALPFLERLLLLHRDS

>sp|Q8TE82|S3TC1_HUMAN/903-936

AVGLANFGALCLHAGASRLAQHYLLEAVRLFSRL

>sp|Q8TE82|S3TC1_HUMAN/1192-1225

RVAYHRLAALQHRLGHGELAEHFYLKALSLCNSP

>sp|Q8TF17|S3TC2_HUMAN/836-869

GVIYNLLGLALQGEGRVNRAAKSYLRALNRAQEV

>sp|Q8TF17|S3TC2_HUMAN/1166-1199

LVAFHRLATVYYSLHMYEMAEDCYLKTLSLCPPW

>sp|Q8TF17|S3TC2_HUMAN/528-561

ARLCFLLGRLSIRKVKLSQARVYFEEAIHILNGA

>sp|Q8TF17|S3TC2_HUMAN/1044-1077

AEAWLGAGRLHYLMQEDELVELCLQAAIQTALKS

>sp|Q8WVT3|TPC12_HUMAN/580-613

PQLLSGIGRISLQIGDIKTAEKYFQDVEKVTQKL

>sp|Q8WVT3|TPC12_HUMAN/654-687

AVANNNAAVCLLYLGKLKDSLRQLEAMVQQDPRH

>sp|Q8WVT3|TPC12_HUMAN/545-578

GRVMYSMANCLLLMKDYVLAVEAYHSVIKYYPEQ

>sp|Q8WVT3|TPC12_HUMAN/620-653

IMVLMNSAFLHLGQNNFAEAHRFFTEILRMDPRN

>sp|Q8WXU2|DAAF4_HUMAN/290-323

PEWLKDKGNKLFATENYLAAINAYNLAIRLNNKM

>sp|Q8WXU2|DAAF4_HUMAN/324-357

PLLYLNRAACHLKLKNLHKAIEDSSKALELLMPP

>sp|Q8WXU2|DAAF4_HUMAN/366-399

MKAHVRRGTAFCQLELYVEGLQDYEAALKIDPSN

>sp|Q92623|TTC9A_HUMAN/57-90

AHEFKSQGAQCYKDKKFREAIGKYHRALLELKGL

>sp|Q92623|TTC9A_HUMAN/164-197

FKALYRSGVAFYHLGDYDKALYYLKEARTQQPTD

>sp|Q92624|APBP2_HUMAN/246-279

VDVLRQASKACVVKREFKKAEQLIKHAVYLARDH

>sp|Q92624|APBP2_HUMAN/429-462

AKHYGNLGRLYQSMRKFKEAEEMHIKAIQIKEQL

>sp|Q96AE7|TTC17_HUMAN/1085-1118

AVNHFTLGNVYVAMEEFEKALVWYESTLKLQPEF

>sp|Q96AE7|TTC17_HUMAN/655-688

DVPLVNLANLLIHYGLHLDATKLLLQALAINSSE

>sp|Q96AE7|TTC17_HUMAN/619-652

WLILNEAGLYWRAVGNSTFAIACLQRALNLAPLQ

>sp|Q96AE7|TTC17_HUMAN/295-328

FTSYYTLGNIYAMLGEYNHSVLCYDHALQARPGF

>sp|Q96AE7|TTC17_HUMAN/689-722

PLTFLSLGNAYLALKNISGALEAFRQALKLTTKC

>sp|Q96AE7|TTC17_HUMAN/1051-1084

DVPLISLANILHNAKLWNDAVIVATMAVEIAPHF

>sp|Q96AE7|TTC17_HUMAN/1015-1048

WVLSSMAALYWRVKGQGKKAIDCLRQALHYAPHQ

>sp|Q96AY4|TTC28_HUMAN/634-667

GKVCHNLGYAHYCLGNYQEAVKYYEQDLALAKDL

>sp|Q96AY4|TTC28_HUMAN/58-91

FVEKVRQSNQACHDGDFHTAIVLYNEALAVDPQN

>sp|Q96AY4|TTC28_HUMAN/474-507

GRASSNLGIIHQMKGDYDTALKLHKTHLCIAQEL

>sp|Q96AY4|TTC28_HUMAN/554-587

ASTHGNLAVAYQALGAHDRALQHYQNHLNIAREL

>sp|Q96AY4|TTC28_HUMAN/1037-1070

GRAYGNLGLTYESLGTFERAVVYQEQHLSIAAQM

>sp|Q96AY4|TTC28_HUMAN/126-159

PKAYFRQGVALQYLGRHADALAAFASGLAQDPKS

>sp|Q96AY4|TTC28_HUMAN/594-627

ARALSNLGNFHCSRGEYVQAAPYYEQYLRLAPDL

>sp|Q96AY4|TTC28_HUMAN/714-747

FRALGNLGDIFICKKDINGAIKFYEQQLGLAHQV

>sp|Q96AY4|TTC28_HUMAN/754-787

ASAYAALGTAYRMIQKYDKALGYHTQELEVYQEL

>sp|Q96AY4|TTC28_HUMAN/997-1030

SDAACGLGGVYQQMGEYDTALQYHQLDLQIAEET

>sp|Q96AY4|TTC28_HUMAN/274-307

CRAHGNLGSAFFSKGNYREALTNHRHQLVLAMKL

>sp|Q96AY4|TTC28_HUMAN/314-347

SSALSSLGHVYTAIGDYPNALASHKQCVLLAKQS

>sp|Q96AY4|TTC28_HUMAN/957-990

AQAYGELGSLHSQLGNYEQAISCLERQLNIARDM

>sp|Q96AY4|TTC28_HUMAN/354-387

ARELGNMGAVYIAMGDFENAVQCHEQHLKIAKDL

>sp|Q96AY4|TTC28_HUMAN/674-707

AKAYCNLGLAFKALLNFSKAEECQKYLLSLAQSL

>sp|Q96AY4|TTC28_HUMAN/1117-1150

AKIRHGLGLSLWASGNLEEAQHQLYRASALFETI

>sp|Q96AY4|TTC28_HUMAN/794-827

CRAHGHLAAVYMALGKYTMAFKCYEEQLDLGQKL

>sp|Q96AY4|TTC28_HUMAN/917-950

AKAYRGLGNGHRAMGSLQQALVCFEKRLVVAHEL

>sp|Q96AY4|TTC28_HUMAN/434-467

MRAYAGLGHAARCMQDLERAKQYHEQQLGIAEDL

>sp|Q96AY4|TTC28_HUMAN/834-867

AQVYGNMGITKMNMNVMEEAIGYFEQQLAMLQQL

>sp|Q96AY4|TTC28_HUMAN/1077-1110

TVSYSSLGRTHHALQNYSQAVMYLQEGLRLAEQL

>sp|Q96AY4|TTC28_HUMAN/877-910

GRAYGNLGDCYEALGDYEEAIKYYEQYLSVAQSL

>sp|Q96AY4|TTC28_HUMAN/514-547

GRAYGNMGNAYNALGMYDQAVKYHRQELQISMEV

>sp|Q96AY4|TTC28_HUMAN/394-427

ARAYSNLGSAYHYRRNFDKAMSYHNYVLELAQEL

>sp|Q96AY4|TTC28_HUMAN/92-125

CILYSNRSAAYMKIQQYDKALDDAIKARLLNPKW

>sp|Q96EQ0|SGTB_HUMAN/85-118

ADQLKDEGNNHMKEENYAAAVDCYTQAIELDPNN

>sp|Q96EQ0|SGTB_HUMAN/119-152

AVYYCNRAAAQSKLGHYTDAIKDCEKAIAIDSKY

>sp|Q96EQ0|SGTB_HUMAN/153-186

SKAYGRMGLALTALNKFEEAVTSYQKALDLDPEN

>sp|Q96HA7|TONSL_HUMAN/202-235

FRARYNLGTIHWRAGQHSQAMRCLEGARECAHTM

>sp|Q96HA7|TONSL_HUMAN/242-275

SECCVVIAQVLQDLGDFLAAKRALKKAYRLGSQK

>sp|Q96HA7|TONSL_HUMAN/311-344

MVICEQLGDLFSKAGDFPRAAEAYQKQLRFAELL

>sp|Q96HA7|TONSL_HUMAN/67-100

AVAHRKIGERLAEMEDYPAALQHQHQYLELAHSL

>sp|Q96HA7|TONSL_HUMAN/352-385

AIIHVSLATTLGDMKDHHGAVRHYEEELRLRSGN

>sp|Q96HA7|TONSL_HUMAN/27-60

AALCHQLGELLAGHGRYAEALEQHWQELQLRERA

>sp|Q96HA7|TONSL_HUMAN/162-195

TRLYLNLGLTFESLQQTALCNDYFRKSIFLAEQN

>sp|Q96N46|TTC14_HUMAN/382-415

CQTLVERGGQLEEEEKFLNAESYYKKALALDETF

>sp|Q96N46|TTC14_HUMAN/341-374

VEALVARGALYATKGSLNKAIEDFELALENCPTH

>sp|Q96N46|TTC14_HUMAN/307-340

ALKCVKIGVDYFKVGRHVDAMNEYNKALEIDKQN

>sp|Q96RK4|BBS4_HUMAN/338-371

GELYMLLAVALTNLEDIENAKRAYAEAVHLDKCN

>sp|Q96RK4|BBS4_HUMAN/270-303

PPLWNNIGMCFFGKKKYVAAISCLKRANYLAPFD

>sp|Q96RK4|BBS4_HUMAN/135-168

WEISHNLGVCYIYLKQFNKAQDQLHNALNLNRHD

>sp|Q96RK4|BBS4_HUMAN/101-134

ADNLKQVARSLFLLGKHKAAIEVYNEAAKLNQKD

>sp|Q96RK4|BBS4_HUMAN/304-337

WKILYNLGLVHLTMQQYASAFHFLSAAINFQPKM

>sp|Q96RK4|BBS4_HUMAN/67-100

EYAIYVQALIFRLEGNIQESLELFQTCAVLSPQS

>sp|Q96RK4|BBS4_HUMAN/202-235

TELLTTLGLLYLQLGIYQKAFEHLGNALTYDPTN

>sp|Q99614|TTC1_HUMAN/116-149

STRLKEEGNEQFKKGDYIEAESSYSRALEMCPSC

>sp|Q99614|TTC1_HUMAN/189-222

IRAILRRAELYEKTDKLDEALEDYKSILEKDPSI

>sp|Q99614|TTC1_HUMAN/155-188

SILFSNRAAARMKQDKKEMAINDCSKAIQLNPSY

>sp|Q99615|DNJC7_HUMAN/96-129

VRGHLREGKCHLSLGNAMAACRSFQRALELDHKN

>sp|Q99615|DNJC7_HUMAN/176-209

HRFKILKAECLAMLGRYPEAQSVASDILRMDSTN

>sp|Q99615|DNJC7_HUMAN/294-327

AKLYCNRGTVNSKLRKLDDAIEDCTNAVKLDDTY

>sp|Q99615|DNJC7_HUMAN/328-361

IKAYLRRAQCYMDTEQYEEAVRDYEKVYQTEKTK

>sp|Q99615|DNJC7_HUMAN/28-61

AETFKEQGNAYYAKKDYNEAYNYYTKAIDMCPKN

>sp|Q99615|DNJC7_HUMAN/210-243

ADALYVRGLCLYYEDCIEKAVQFFVQALRMAPDH

>sp|Q99615|DNJC7_HUMAN/62-95

ASYYGNRAATLMMLGRFREALGDAQQSVRLDDSF

>sp|Q99615|DNJC7_HUMAN/256-289

LKAKKEDGNKAFKEGNYKLAYELYTEALGIDPNN

>sp|Q99666|RGPD5_HUMAN/60-93

PKAHRFLGLLYELEENTEKAVECYRRSVELNPTQ

>sp|Q9BXJ9|NAA15_HUMAN/408-441

IELFLVKAKIYKHAGNIKEAARWMDEAQALDTAD

>sp|Q9BXJ9|NAA15_HUMAN/374-407

LWVQYYLAQHYDKIGQPSIALEYINTAIESTPTL

>sp|Q9BXJ9|NAA15_HUMAN/46-79

GETLAMKGLTLNCLGKKEEAYELVRRGLRNDLKS

>sp|Q9BXJ9|NAA15_HUMAN/485-518

MWFQTECAQAYKAMNKFGEALKKCHEIERHFIEI

>sp|Q9BXJ9|NAA15_HUMAN/224-257

LAVEETKGELLLQLCRLEDAADVYRGLQERNPEN

>sp|Q9BXJ9|NAA15_HUMAN/80-113

HVCWHVYGLLQRSDKKYDEAIKCYRNALKWDKDN

>sp|Q9C0D5|TANC1_HUMAN/1289-1322

LQKLMEEGNVMYKKGKMKEAAQRYQYALRKFPRE

>sp|Q9C0D5|TANC1_HUMAN/1370-1403

YEAFYARARAKRNSRQFVAALADLQEAVKLCPTN

>sp|Q9C0D5|TANC1_HUMAN/1336-1369

VSLYLNLSRCRRKTNDFGMAEEFASKALELKPKS

>sp|Q9H0B6|KLC2_HUMAN/366-399

AKTKNNLASCYLKQGKYQDAETLYKEILTRAHEK

>sp|Q9H0B6|KLC2_HUMAN/282-315

AATLNNLAVLYGKRGKYKEAEPLCKRALEIREKV

>sp|Q9H0B6|KLC2_HUMAN/240-273

ATMLNILALVYRDQNKYKEAAHLLNDALAIREKT

>sp|Q9H0B6|KLC2_HUMAN/324-357

AKQLSNLALLCQNQGKAEEVEYYYRRALEIYATR

>sp|Q9H3U1|UN45A_HUMAN/21-54

VEQLRKEGNELFKCGDYGGALAAYTQALGLDATP

>sp|Q9H3U1|UN45A_HUMAN/58-91

AVLHRNRAACHLKLEDYDKAETEASKAIEKDGGD

>sp|Q9H3U1|UN45A_HUMAN/92-125

VKALYRRSQALEKLGRLDQAVLDLQRCVSLEPKN

>sp|Q9H6T3|RPAP3_HUMAN/350-383

SKAFARRGTARTFLGKLNEAKQDFETVLLLEPGN

>sp|Q9H6T3|RPAP3_HUMAN/133-166

ALVLKEKGNKYFKQGKYDEAIDCYTKGMDADPYN

>sp|Q9H6T3|RPAP3_HUMAN/167-200

PVLPTNRASAYFRLKKFAVAESDCNLAVALNRSY

>sp|Q9H6T3|RPAP3_HUMAN/316-349

ALLPANRAMAYLKIQKYEEAEKDCTQAILLDGSY

>sp|Q9H6T3|RPAP3_HUMAN/201-234

TKAYSRRGAARFALQKLEEAKKDYERVLELEPNN

>sp|Q9H6T3|RPAP3_HUMAN/282-315

AISEKDRGNGFFKEGKYERAIECYTRGIAADGAN

>sp|Q9H892|TTC12_HUMAN/174-207

TKAYFHMGKANLALKNYSVSRECYKKILEINPKL

>sp|Q9H892|TTC12_HUMAN/140-173

KVLYTNRAQAYMKLEDYEKALVDCEWALKCDEKC

>sp|Q9H892|TTC12_HUMAN/106-139

ADALKEKGNEAFAEGNYETAILRYSEGLEKLKDM

>sp|Q9H9A5|CNO10_HUMAN/355-388

YELLYNCGIQLLHIGRPLAAFECLIEAVQVYHAN

>sp|Q9H9A5|CNO10_HUMAN/643-676

TVMLFNLGSAYCLRSEYDKARKCLHQAASMIHPK

>sp|Q9H9A5|CNO10_HUMAN/110-143

SMLYYNQAVILYHLRQYTEAISVGEKLYQFIEPF

>sp|Q9H9A5|CNO10_HUMAN/293-326

CMFWNNLGCIHFAMSKHNLGIFYFKKALQENDNV

>sp|Q9HCD6|TANC2_HUMAN/1291-1324

VSLLLNLSRCRRKMNDFGMAEEFATKALELKPKS

>sp|Q9HCD6|TANC2_HUMAN/1244-1277

LSKLMEEGDMFYKKGKVKEAAQRYQYALKKFPRE

>sp|Q9HCD6|TANC2_HUMAN/1325-1358

YEAYYARARAKRSSRQFAAALEDLNEAIKLCPNN

>sp|Q9HCS7|SYF1_HUMAN/256-289

GKLWCSLADYYIRSGHFEKARDVYEEAIRTVMTV

>sp|Q9HCS7|SYF1_HUMAN/395-428

HTLWVAFAKFYEDNGQLDDARVILEKATKVNFKQ

>sp|Q9HCS7|SYF1_HUMAN/433-466

ASVWCQCGELELRHENYDEALRLLRKATALPARR

>sp|Q9NSK0|KLC4_HUMAN/464-497

NTTLRNLGALYRRQGKLEAAETLEECALRSRRQG

>sp|Q9NSK0|KLC4_HUMAN/295-328

AATLNNLAVLYGKRGKYKEAEPLCQRALEIREKV

>sp|Q9NSK0|KLC4_HUMAN/253-286

ATMLNILALVYRDQNKYKEAAHLLNDALSIREST

>sp|Q9NSK0|KLC4_HUMAN/379-412

ARTKNNLASCYLKQGKYAEAETLYKEILTRAHVQ

>sp|Q9NSK0|KLC4_HUMAN/337-370

AKQLNNLALLCQNQGKYEAVERYYQRALAIYEGQ

>sp|Q9NZN9|AIPL1_HUMAN/264-297

VKAYYVRARAHAEVWNEAEAKADLQKVLELEPSM

>sp|Q9NZN9|AIPL1_HUMAN/178-211

VPVLHGEGNRLFKLGRYEEASSKYQEAIICLRNL

>sp|Q9UGR2|Z3H7B_HUMAN/116-149

IRALFRKARALNELGRHKEAYECSSRCSLALPHD

>sp|Q9UGR2|Z3H7B_HUMAN/82-115

CKLHVNRAACYFTMGLYEKALEDSEKALGLDSES

>sp|Q9UIM3|FKBPL_HUMAN/210-243

AREERARGTELFRAGNPEGAARCYGRALRLLLTL

>sp|Q9UIM3|FKBPL_HUMAN/286-319

LKALYRRGVAQAALGNLEKATADLKKVLAIDPKN

>sp|Q9UIM3|FKBPL_HUMAN/252-285

TVLHANLAACQLLLGQPQLAAQSCDRVLEREPGH

>sp|Q9UJX2|CDC23_HUMAN/331-364

VETCCVIGNYYSLRSQHEKAALYFQRALKLNPRY

>sp|Q9UJX2|CDC23_HUMAN/263-296

SYIVSQIAVAYHNIRDIDKALSIFNELRKQDPYR

>sp|Q9UJX2|CDC23_HUMAN/433-466

SRMLVALGECYEKLNQLVEAKKCYWRAYAVGDVE

>sp|Q9UJX2|CDC23_HUMAN/399-432

YRAWYGLGQTYEILKMPFYCLYYYRRAHQLRPND

>sp|Q9UJX2|CDC23_HUMAN/365-398

LGAWTLMGHEYMEMKNTSAAIQAYRHAIEVNKRD

>sp|Q9UJX2|CDC23_HUMAN/510-543

STAFRYLAQYYFKCKLWDEASTCAQKCCAFNDTR

>sp|Q9UJX2|CDC23_HUMAN/169-202

GFGLYLYGVVLRKLDLVKEAIDVFVEATHVLPLH

>sp|Q9UJX3|APC7_HUMAN/373-406

VQALLLKGAALRNMGRVQEAIIHFREAIRLAPCR

>sp|Q9UJX3|APC7_HUMAN/169-202

PKINMMLANLYKKAGQERPSVTSYKEVLRQCPLA

>sp|Q9UJX3|APC7_HUMAN/509-542

CVLHRILGDFLVAVNEYQEAMDQYSIALSLDPND

>sp|Q9UJX3|APC7_HUMAN/271-304

VDLLGSLADLYFRAGDNKNSVLKFEQAQMLDPYL

>sp|Q9UJX3|APC7_HUMAN/339-372

AEPWVVSGCHSFYSKRYSRALYLGAKAIQLNSNS

>sp|Q9UJX4|APC5_HUMAN/467-500

AVALCHLAELHAEQGCFAAASEVLKHLKERFPPN

>sp|Q9UJX4|APC5_HUMAN/301-334

RYAALNLAALHCRFGHYQQAELALQEAIRIAQES

>sp|Q9UJX4|APC5_HUMAN/581-614

ISVLLSVAELYWRSSSPTIALPMLLQALALSKEY

>sp|Q9UJX4|APC5_HUMAN/541-574

IEGVYRKAVVLQAQNQMSEAHKLLQKLLVHCQKL

>sp|Q9ULT0|TTC7A_HUMAN/566-599

AHALHLLALLFSAQKHHQHALDVVNMAITEHPEN

>sp|Q9ULT0|TTC7A_HUMAN/414-447

FHLWYQVALSMVACGKSAYAVSLLRECVKLRPSD

>sp|Q9ULT0|TTC7A_HUMAN/813-846

HEAWQGLGEVLQAQGQNEAAVDCFLTALELEASS

>sp|Q9ULT0|TTC7A_HUMAN/745-778

HSVLYMRGRLAEVKGNLEEAKQLYKEALTVNPDG

>sp|Q9ULT0|TTC7A_HUMAN/711-744

EQIWLQAAELFMEQQHLKEAGFCIQEAAGLFPTS

>sp|Q9ULT0|TTC7A_HUMAN/779-812

VRIMHSLGLMLSRLGHKSLAQKVLRDAVERQSTC

>sp|Q9UNE7|CHIP_HUMAN/60-93

AVYYTNRALCYLKMQQHEQALADCRRALELDGQS

>sp|Q9UNE7|CHIP_HUMAN/94-127

VKAHFFLGQCQLEMESYDEAIANLQRAYSLAKEQ

>sp|Q9UNE7|CHIP_HUMAN/26-59

AQELKEQGNRLFVGRKYPEAAACYGRAITRNPLV

>sp|Q9Y2Z0|SGT1_HUMAN/45-78

AQYYCQRAYCHILLGNYCVAVADAKKSLELNPNN

>sp|Q9Y2Z0|SGT1_HUMAN/79-112

STAMLRKGICEYHEKNYAAALETFTEGQKLDIET

>sp|Q9Y5Q9|TF3C3_HUMAN/217-250

TEEWVRLAEMSLEQDNIKQAIFCYTKALKYEPTN

>sp|Q9Y5Q9|TF3C3_HUMAN/811-844

QESFYNLGRGLHQLGLIHLAIHYYQKALELPPLV

>sp|Q9Y5Q9|TF3C3_HUMAN/251-284

VRYLWERSSLYEQMGDHKMAMDGYRRILNLLSPS

>sp|Q9Y5Q9|TF3C3_HUMAN/456-489

AVVWLRHAECLKALGYMERAAESYGKVVDLAPLH

>sp|Q9Y5Q9|TF3C3_HUMAN/183-216

YEPFSTLAMIYEDQGDMEKSLQFELIAAHLNPSD

>sp|Q9Y5Q9|TF3C3_HUMAN/149-182

LRGLMGEANIRFARGEREEAILMCMEIIRQAPLA

>sp|Q9Y6J0|CABIN_HUMAN/1055-1088

NELYYLLADYHFKNKEQSKAIKFYMHDICICPNR

>sp|Q9Y6J0|CABIN_HUMAN/615-648

VRVYWLKARFLALQGDMEQALENYDICTEMLQSS

>sp|Q9Y6J0|CABIN_HUMAN/90-123

YSTYKNLAQLAAQREDLETAMEFYLEAVMLDSTD

>sp|Q9Y6J0|CABIN_HUMAN/124-157

VNLWYKIGHVALRLIRIPLARHAFEEGLRCNPDH

>sp|Q9Y6J0|CABIN_HUMAN/36-69

AFALYHKALDLQKHDRFEESAKAYHELLEASLLR

>sp|Q9Y6X3|SCC4_HUMAN/107-140

FEAASLLSELYCQENSVDAAKPLLRKAIQISQQT

>sp|Q9Y6X3|SCC4_HUMAN/379-412

AQLHTLLGLYCVSVNCMDNAEAQFTTALRLTNHQ

>sp|Q9Y6X3|SCC4_HUMAN/459-492

AAAFYVRGLFSFFQGRYNEAKRFLRETLKMSNAE
